# Influenza A Virus Facilitates Its Infectivity by Activating p53 to Inhibit the Expression of Interferon-Induced Transmembrane Proteins

**DOI:** 10.3389/fimmu.2018.01193

**Published:** 2018-05-31

**Authors:** Bei Wang, Tze Hau Lam, Mun Kuen Soh, Zhiyong Ye, Jinmiao Chen, Ee Chee Ren

**Affiliations:** ^1^Singapore Immunology Network, Agency for Science, Technology and Research (A*STAR), Singapore, Singapore; ^2^Department of Microbiology and Immunology, National University of Singapore, Singapore, Singapore

**Keywords:** influenza A virus, p53, IFITM1, IFITM2, IFITM3, infectivity, antiviral, CRISPR/Cas9

## Abstract

Human influenza virus (IAV) are among the most common pathogens to cause human respiratory infections. A better understanding on interplay between IAV and host factors may provide clues for disease prevention and control. While many viruses are known to downregulate p53 upon entering the cell to reduce the innate host antiviral response, IAV infection is unusual in that it activates p53. However, it has not been clear whether this process has proviral or antiviral effects. In this study, using human isogenic p53 wild-type and p53null A549 cells generated from the CRISPR/Cas9 technology, we observed that p53null cells exhibit significantly reduced viral propagation when infected with influenza A virus (strain A/Puerto Rico/8/1934 H1N1). Genome-wide microarray analysis revealed that p53 regulates the expression of a large set of interferon-inducible genes, among which the interferon-induced transmembrane family members IFITM1, IFITM2, and IFITM3 were most significantly downregulated by the expression of p53. Knockdown of interferon-induced transmembrane proteins (IFITMs) by short interfering RNAs enhanced influenza virus infectivity in p53null A549 cells, while overexpressed IFITMs in A549 cells blocked virus entry. Intriguingly, regulation of IFITMs by p53 is independent of its transcriptional activity, as the p53 short isoform Δ40p53 recapitulates IFITM regulation. Taken together, these data reveal that p53 activation by IAV is an essential step in maintaining its infectivity. This novel association between human p53 and the broad spectrum antiviral proteins, the IFITMs, demonstrates a previous mechanism employed by influenza virus to enhance its propagation *via* p53 inhibition of IFITMs.

## Introduction

Human influenza virus (IAV) infection poses a global health threat but the extent of disease severity varies greatly between individuals, suggesting that host factors play an important role in pathogenesis of influenza virus infection (1). Of particular interest in IAV infection is the interferon-induced transmembrane protein (IFITM) family, which was first identified over 20 years ago (2), and later revealed to restrict infectivity of diverse viral pathogens, including IAV (3), dengue, rabies, Ebola (4), and reovirus (5). Specifically, IFITM3 can restrict IAV infection in the mouse (6, 7), and a SNP variant in human IFITM3 has been linked with more severe symptoms of influenza virus infection (7). Although the mechanisms by which IFITMs inhibit virus infection are not fully elucidated, there is evidence that IFITMs may block virus infectivity by interfering with virus–endosome fusion, and by restricting IAV exit from late endosomes *via* blocking fusion pore formation (3, 8).

Upon entering the cells, many viruses are known to downregulate p53, a key component of the cellular stress machinery and the host anti-IAV response (9), However, influenza virus is unusual in that it activates cellular p53 (10). p53 has been reported to promote apoptotic cell death in IAV-infected cells (10), as well as enhancing the type I interferon pathway and production of associated molecules in mouse model (11), and boosting the *in vivo* antiviral DC and T cell responses (11). Antiviral effects of p53 during IAV infection has been suggested (10), and in mouse models, viral load was found to be significantly higher in *p53*^−/−^ than *p53*^+/+^ mice during influenza virus infection (11). However, there is also some evidence suggesting proviral effects of p53 during IAV infection which found a positive correlation between p53 upregulation and viral protein levels (12–14).

Here, we asked how p53 status affected transcription of antiviral genes, and how these factors combined to determine cellular susceptibility to IAV infection. We uncovered a direct link between p53 expression during IAV infection and reduced IFITM induction, leading to increased IAV propagation in human epithelial cell lines. Thus, IAV exploits cellular p53 to prevent high level expression of IFITMs that would otherwise block viral infection spreading through the local cell population.

In this study, by generating p53null lung epithelial cell lines from A549 cells carrying wild-type p53 alleles using the new genome-editing technology CRISPR/Cas9 (15), we were, for the first time, able to compare in parallel the response to influenza infection in an isogenic background of p53WT and p53null human cells that are permissive to influenza virus infection. p53null cells displayed attenuated influenza virus propagation compared with p53WT cells. Using genome-wide expression profile screens, we identified IFITM1, IFITM2, and IFITM3 as downstream molecules of p53 pathway and were negatively associated with p53. Rescue assays demonstrated that high level of IFITMs in p53null cells confer the resistance to influenza virus infection. Taken together, these data suggest that influenza virus maintain its infectivity by harnessing cellular p53 to restrict the antiviral protein family IFITMs.

## Materials and Methods

### Cell Lines and Viruses

The human lung carcinoma cell line A549 (CCL-185) was purchased from ATCC (Manassas, VA, USA). All cells were maintained in Dulbecco’s modified Eagle’s medium supplemented with 10% fetal bovine serum (Thermo Fisher Scientific, Waltham, MA, USA) and cultured in a 37°C incubator with 5% CO_2_.

### Virus and Viral Infection

Human influenza A virus stock (strain A/Puerto Rico/8/1934 H1N1) propagated in specific pathogen free chicken eggs was kindly provided by Dr. Vincent Chow (National University of Singapore). Parental A549 cells and their CRISPR/Cas9-generated p53null clones were plated onto cell culture plates or chamber slides (μ-Slide VI 0.4, ibidi GmbH, Planegg, Germany) and after overnight incubation, washed with sterile PBS twice, and then infected with influenza A virus at a multiplicity of infection (MOI) of 0.0025–0.008 in virus inoculation medium (DMEM containing 25 mM HEPES). After 1.5 h adsorption, the inocula were removed, and the cells were cultured in virus maintenance medium (DMEM containing 25 mM HEPES and 1% BSA) for various time points.

### CRISPR/Cas9-Mediated p53 Knockout

Single-guide RNA (sgRNA) sequences targeting p53 (sgRNA-a-F: 5′-CACCGGGCAGCTACGGTTTCCGTC-3′, sgRNA-a-R: 5′-AAACGACGGAAACCGTAGCTG-CCC-3′, sgRNA-b-F: 5′-CACCGAGCGCTGCTCAGATAGCGA-3′, sgRNA-b-R: 5′-AAACTCGCTATCTGAGCAGCGCTC-3′, sgRNA-c-F: 5′-CACCGTCGACGCTAGGATCT-GACTG-3′, sgRNA-c-R: 5′-AAACCAGTCAGATCCTAGCGTCGAC-3′) were cloned into the Bbs1 site of the pX330 plasmid (Addgene, Cambridge, MA, USA) by simple annealing and ligation. A549 cells were transfected with different sgRNA constructs using Lipofectamine 2000 reagent (Thermo Fisher Scientific), and 24 h later were selected for 1 week in medium with Nutlin-3 (Final concentration: 40 µM, Sigma-Aldrich, St. Louis, MO, USA), a Mdm2 (Mouse double minute 2 homolog) antagonist that activates p53 and induce cellular apoptosis. Surviving cells were trypsinized and re-plated on Terasaki plates (Grenier Bio-One, Kremsmünster, Austria) in single cell suspension by limiting dilution and cultured for 12–14 days to form cell colonies. Single cell colonies were picked, expanded, and assayed for p53 expression using Western blot with p53 antibodies (sc-126 or sc-6243, Santa Cruz Biotechnology, Dallas, TX, USA). Another p53-activating reagent 5-fluorouracil was purchased from Sigma-Aldrich and reconstituted in DMSO.

### Caspase 3/7 Assay, Cytotoxicity Assay, and Viability Assay

The cytotoxicity assay, cell viability assay, and caspase 3/7 assays were carried out in a single well at 24 h post-IAV infection using ApoTox-Glo™ Triplex Assay (Promega Corporation, Madison, WI, USA) according to the manufacturer’s standard protocol. Caspase 3-specific inhibitor Z-DEVD-fmk was purchased from Santa Cruz Biotechnology (sc-311558), reconstituted in sterile DMSO to 2 mM stock solution, diluted to 40 µM working concentration with virus maintenance medium, and added to the cells 1.5 h after virus adsorption.

### RNA Isolation and Real-Time Quantitative PCR (RT-qPCR) Analysis

Total RNA of experimental cells was extracted by RNeasy Mini Kit (Qiagen, Hilden, Germany) with in-column DNase digestion using RNase-free DNase Set (Qiagen). The viral RNA from culture supernatant was isolated by QIAamp Viral RNA Mini Kit (Qiagen). After quantification by Nanodrop, cDNA was synthesized by Maxima First Strand cDNA Synthesis Kit (Fermentas, Thermo Fisher Scientific). mRNA expression of different genes was analyzed by RT-qPCR using KAPA SYBR FAST qPCR Kit (KAPA Biosystems, Wilmington, MA, USA) on LightCycler 480 Instrument (Roche Diagnostics, Risch-RotKreuz, Switzerland). HPRT was used as a reference gene. RT-qPCR data were analyzed using the standard 2^−ΔΔCT^ method (16) and presented as the fold expression normalized to the reference HPRT gene. All primers used for RT-qPCR are listed in Supplementary Material.

### Western Blot

Cells were lysed in 1% Nonidet P-40 cell lysis buffer containing the complete mini protease inhibitor cocktail (Roche Diagnostics) on ice for 30 min and the protein concentration was determined using standard Bradford Protein Assay (Bio-Rad Laboratories, Hercules, CA, USA). 5–10 µg of cell lysate was separated by 12.5% SDS-PAGE and transferred to Hybond PVDF membranes (GE Healthcare, Little Chalfont, UK). Antibodies against p53 (DO-1, sc-126, 1:3,000), full-length p53 (FL393, sc-6243, 1:500), p21 (C-19, sc-397, 1:250) (Santa Cruz Biotechnology), IFITM1 (60074-1-Ig, 1:1,000), IFITM2/3 (66081-1-Ig, 1:7,000) (Proteintech Group, Inc., Rosemont, IL, USA), and PR8 NP (GTX125989, 1:5,000, GeneTex, Inc., Irvine, CA, USA) were used as primary antibodies. HRP-conjugated donkey-anti-mouse IgG or goat-anti-rabbit IgG (Thermo Fisher Scientific) were applied as secondary antibodies for protein detection. Equal loading of protein samples was verified with an antibody against β-actin (1:20,000, Merck Millipore, Burlington, MA, USA), and the immunoreactive signals were visualized using enhanced chemiluminescence reagents (GE Healthcare).

### IFITMs Cloning

Molecular cloning of IFITM1, IFITM2, and IFITM3 full coding sequences to pcDNA3.1 (+) plasmid (Thermo Fisher Scientific) was carried out using the In-Fusion^®^ HD Cloning kit (Clontech Laboratories, Mountain View, CA, USA) according to the manufacturer’s instructions. The primers were designed to include the BamH I and Xho I sites and additional 10-bp sequences spanning the enzyme cut site homologous to vector sequences. All primers used for cloning are listed in Supplementary Material.

### Short Interfering RNAs (siRNAs) and siRNA Transfection

Pre-designed pooled siRNAs targeting IFITM1 (si-IFITM1), IFITM2 (si-IFITM2), and IFITM3 (si-IFITM3), as well as negative control scramble siRNA (si-Ctrl) were purchased from Dharmacon (Lafayett, CO, USA). Cells were transfected with different siRNAs using Lipofectamine 2000 reagent (Thermo Fisher Scientific).

### Flow Cytometry Analysis

At 24 h post-IAV infection, both mock and infected cells were dissociated by trypsinization using 0.05% Trypsin–EDTA (Thermo Fisher Scientific) and washed once with PBS. Then, cells were thoroughly resuspended in 200 µl of Cytofix/Cytoperm solution (BD Biosciences, San Jose, CA, USA), incubated for 20 min, and washed with 1× Perm/Wash solution (BD Biosciences) before labeling with an anti-IAV nucleoprotein (NP) antibody conjugated to FITC (1:400, GTX36902, GeneTex, Inc.) for 20 min at room temperature. Cells were washed with 1× Perm/Wash solution again and resuspended in 200 µl of PBS for flow cytometry analysis on a LSRII 4-laser flow cytometer (BD Biosciences). All results were analyzed by FlowJo (FlowJo, LLC., Ashland, OR, USA).

### Immunofluorescence Microscopy

Cells were directly seeded onto the chamber wells of μ-Slide VI 0.4 (ibidi GmbH) and after overnight incubation, infected with IAV (strain A/Puerto Rico/8/1934 H1N1) at MOI = 0.001. At 24 h post-infection, cells were fixed in 4% paraformaldehyde (Electron Microscopy Sciences, Hatfield, PA, USA) for 10 min, permeabilized in 0.1% Triton-X100 for 5 min, and blocked with PBS containing 1% bovine serum albumin for at least 1 h before incubating with antibodies specific for IAV NP, conjugated to FITC (1:400, GTX36902, GeneTex) for 1 h then Hoechst 33342 (4 µg/ml, Sigma-Aldrich) for 30 min, before finally mounting with 100 µl Fluorsave mounting medium (Calbiochem, San Diego, CA, USA). Cells were washed with PBS five times between each step. For NP and p53 double staining as well as NP and IFITMs double staining, the cells were first labeled with either mouse-anti-p53 (sc-126, Santa Cruz Biotechnology), or IFITM1 (60074-1-Ig, Proteintech Group), or IFITM2/3 (66081-1-Ig, Proteintech Group Inc.), followed by secondary antibody donkey-anti-mouse-AF546 (1:400, A10036, Life Technologies, Thermo Fisher Scientific), and finally with IAV NP antibody conjugated to FITC (1:400, GTX36902, GeneTex). The fluorescence signals were observed and captured using an Axiovert 200 inverted fluorescence microscope (Carl Zeiss AG, Oberkochen, Germany) or the confocal laser scanning microscope Fluoview FV1000 series (Olympus Corporation, Tokyo, Japan).

### Whole-Genome Microarray Analysis

A549 and A549-KO3 cells were seeded in 12-well plates for overnight culture and then infected with IAV (strain A/Puerto Rico/8/1934 H1N1) at MOI = 0.001 or mock control, each in triplicate. After 24 h of infection, total RNA was extracted with RNeasy Mini Kit (Qiagen), and the genomic DNA was removed by in-column digestion using RNase-free DNase Set (Qiagen). 100 ng of total RNA from each sample was processed by Origen Laboratories Pte Ltd. (Singapore) using Affymetrix GeneChip WT PLUS Reagent Kit (Thermo Fisher Scientific) according to standard Affymetrix recommended protocols, and hybridized to the Affymetrix Human Transcriptome 2.0 Array (Affymetrix, Santa Clara, CA, USA). The HTA 2.0 microarray data were normalized using the sst-rma approach implemented in The Affymetrix^®^ Expression Console™ Software (version 1.4.1.46) for gene expression analysis in log2 scale. Only protein-coding genes were considered for the subsequent downstream evaluation. Differential gene expression between conditions was evaluated by Student’s *t*-test, and the *p*-values were adjusted with Benjamini and Hochberg. Significantly differentially expressed genes were identified with adjusted *p*-value <0.05 and |log2(FC)| > 0.5. All the above statistical analysis was implemented using R version 3.1.1. The microarray data are available at Gene Expression Omnibus with an accession number GSE106279.

### Type I Interferon Treatment

Human recombinant interferon-α1 (IFN-α1, #8927) was purchased from Cell Signaling Technology (Danvers, MA, USA) and reconstituted in sterile PBS to 50 µg/ml stock solution. Human recombinant interferon-β 1a (IFN-β-1a, 11415-1) was from R&D systems (Minneapolis, MN, USA) and reconstituted in sterile PBS to 100,000 U/ml stock solution. Cells were plated on 12-well plates and incubated at 37°C overnight before treatment with different concentrations of IFN-α1 (5 or 10 ng/ml) or IFN-β-1a (50 or 1,000 U/ml) for 24 h.

### Statistical Analyses

All experiments were repeated at least three times with similar results achieved. For most figures, representative images or data are shown. At least triplicates were carried out for each individual test, and data were presented as mean ± SD for each point. Differences between averages or percentages between control sample and tests were statistically analyzed using Wilcoxon–Mann–Whitney test (17). *p*-Values less than 0.05 were considered statistically significant.

## Results

### Generation of p53null Cells Using CRISPR/Cas9

**Figure 1:**
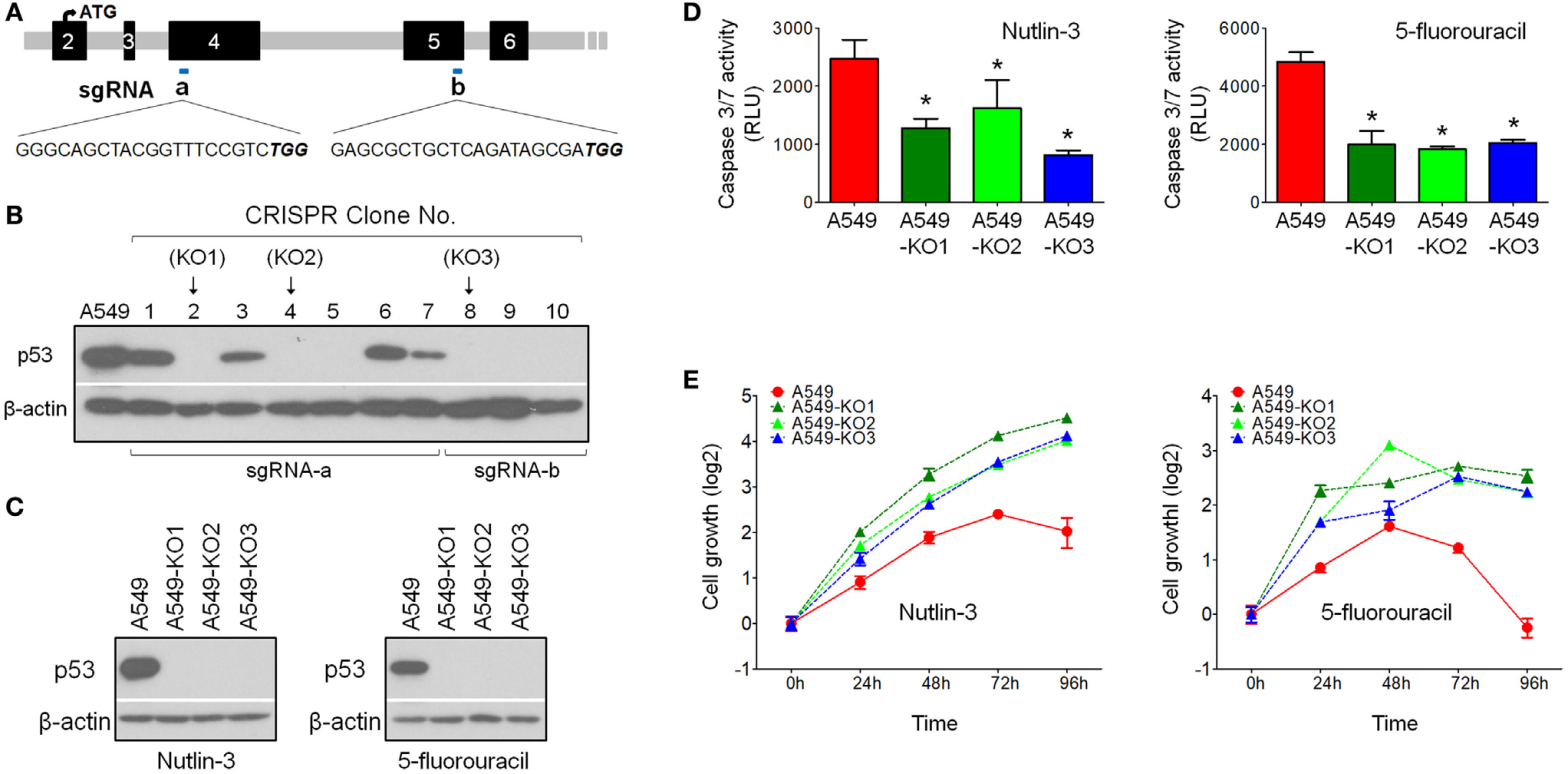
Generation of p53null A549 cells by CRISPR/Cas9 technology. **(A)** Single-guide RNA (sgRNA) sequence designed to target p53WT for gene knockout by CRISPR/Cas9 technology. **(B)** Western blot screening of p53 protein expression in CRISPR A549 cell clones. **(C)** Western blot analysis confirmed loss of p53 protein expression in p53null cell clones A549-KO1, A549-KO2, and A549-KO3. Cells were pretreated with either Nutlin-3 or 5-fluorouracil to induce p53 accumulation in A549 cells. **(D)** Caspase 3/7 assay indicated that all p53null CRISPR clones had lower caspase activity compared with p53WT A549 cells in response to 4 h treatment of Nutlin-3 (25 µM, left panel) or 5-fluorouracil (50 µg/ml, right panel). *p < 0.05. **(E)** WST-1 cell proliferation assay indicated that the three selected p53null CRISPR clones were resistant to Nutlin-3 (15 µM, left panel) or 5-fluorouracil (30 µg/ml, right panel) induced growth inhibition/cell death.

To investigate the role of p53 in influenza virus infectivity, we used the genome-editing technique CRISPR/Cas9-mediated knockout (15) to generate isogenic p53null cell clones from the influenza virus-susceptible lung cancer cell line A549, which carries the wild-type p53 alleles and is also widely used as a model cell line for influenza research. Two sgRNA sequences were designed using the online CRISPR design tool (http://crispr.mit.edu/), targeting exons 4 and 5, respectively (Figure 1A). Ten cell clones were screened using Western blot analysis, showing that six clones had lost p53 protein expression (clones 2, 4, 5, 8, 9, and 10) (Figure 1B). We randomly selected three clones for further analysis: A549-KO1 (clone 2), A549-KO2 (clone 4), and A549-KO3 (clone 8). Sequencing analysis showed that all three clones had DNA modifications in the TP53 locus, adjacent to the sgRNA targeting sites, with either small in-dels formation (A549-KO1 and A549-KO2; Figures S1A,B in Supplementary Material) or a long stretch of nucleotide deletions (A549-KO3; Figure S1C in Supplementary Material). These cell clones displayed characteristic p53null cell properties, having lost p53 protein expression (Figure 1C), as well as exhibiting reduced caspase 3/7 activity (Figure 1D) and resistance to cell death (Figure 1E) in response to the p53-activating drugs Nutlin-3 or 5-fluorouracil.

### p53null Cells Exhibit Attenuated Influenza Virus Propagation Compared With Isogenic Parental p53WT Cells

**Figure 2:**
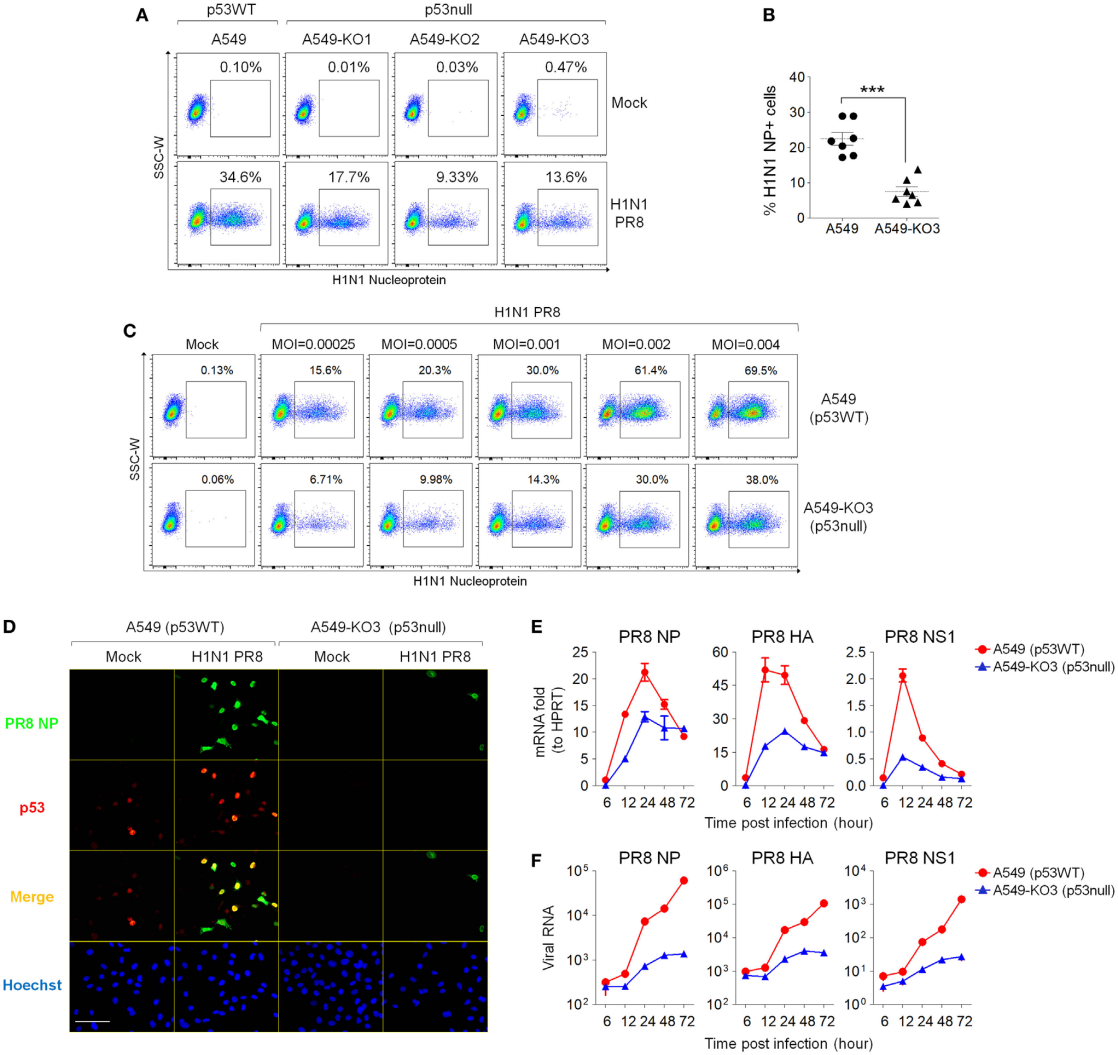
p53null A549 cells exhibit decreased influenza virus susceptibility compared with parental p53WT A549 cells. **(A)** p53null cells A549-KO1, A549-KO2, and A549-KO3 and p53WT A549 were infected with human influenza virus (IAV) (strain A/Puerto Rico/8/1934 H1N1), 24 h later, cells were fixed, permeabilized, and labeled for influenza nucleoprotein (NP) detection by flow cytometry. The population of NP-positive cells was gated, and their percentages are shown. **(B)** Percentages of NP-positive cells 24 h post-IAV infection were compared between A549 and A549-KO3 cells from seven individual experiments. ***p < 0.001. **(C)** Flow cytometry analysis of NP-positive p53WT A549 and A549-KO3 cells 24 h post-IAV infection at a range of multiplicity of infection (MOI). **(D)** Fluorescence imaging of A549 and A549-KO3 cells co-labeled for viral NP (green) and cellular p53 (red) 24 h post-IAV infection. Scale bar, 50 µm. **(E)** Real-time quantitative PCR (RT-qPCR) measurement of viral RNA encoding NP, hemagglutinin (HA), and non-structural protein 1 (NS1) in mock- and IAV-infected A549 and A549-KO3 cells at various time points. **(F)** RT-qPCR measurement of viral RNA encoding NP, HA, and NS1 in culture supernatants of IAV-infected A549 and A549-KO3 cells at various time points.

As influenza virus infection activates cellular p53 (10), we asked what effect p53 has on IAV infection in the A549 model system. To test this, we infected p53WT A549, A549-KO1, A549-KO2, and A549-KO3 cells with IAV (strain A/Puerto Rico/8/1934 H1N1) for 24 h, and then measured the proportion of cells expressing the viral NP using flow cytometry. Here, a MOI of 0.001 was used to mimic physiological conditions for assaying both infectivity and viral replication. All three p53null cell cultures showed consistently lower percentages of viral NP-positive cells (9.33–17.7%) compared with p53WT A549 cells (34.6%) (Figure 2A), suggesting that p53 is necessary for effective infection of A459 cell cultures. We collated the results from individual repeat experiments (MOI = 0.001) using A549 and A549-KO3 cells and calculated for *p*-value of seven experiments as 0.000291 using the Wilcoxon–Mann–Whitney test, demonstrating that p53WT cells had significantly higher level of influenza virus propagation than the representative p53null cells A549-KO3 (Figure 2B). The same pattern was evident across a range of different MOI from 0.00025 to 0.004 (Figure 2C). Consistent with the flow cytometry data, fluorescence imaging showed a higher percentage of IAV-infected cells expressing NP proteins in p53WT A549 cells compared with p53null A549-KO3 cells (Figure 2D; Figures S2A,B in Supplementary Material). In addition, in p53WT A549 cells, p53 was strongly stained and co-localized with viral NP (Figure 2D; Figure S2C in Supplementary Material), further confirming the previous report that influenza virus infection activates cellular p53 (10). Comparable results were seen across all three p53null cell lines (Figures S2D,E in Supplementary Material), clearly showing that p53WT A549 cells were significantly more susceptible to IAV infection than A549-KO1, A549-KO2, and A549-KO3 cells. We also collected cellular RNA and cell culture supernatants to quantify the amount of viral RNA genomes at various time points post-infection using RT-qPCR. In agreement with the flow cytometry and microscopy data, we saw that p53WT A549 cells contained markedly more viral RNAs encoding NP, hemagglutinin (HA), and non-structural protein 1 (NS1), both in live cell lysates (Figure 2E) and in culture supernatants (Figure 2F). Taken together, these data support the observation that p53 is necessary for efficient infection of A459 cells by IAV.

### p53 Does Not Affect Initial IAV Entry Into A459 Cells

**Figure 3:**
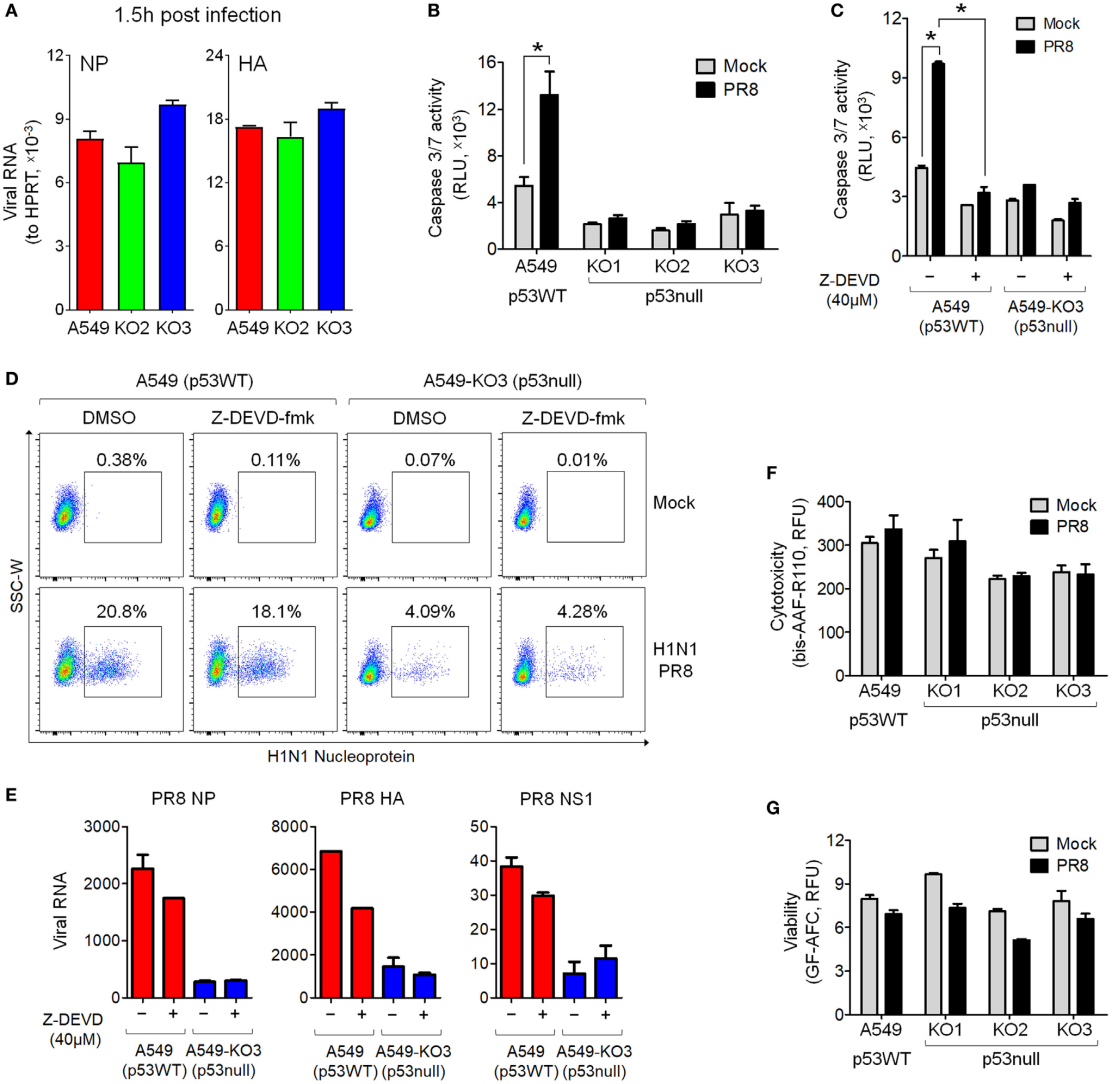
Decreased IAV susceptibility is not mediated by blocked cell entry at the initial stage of infection or decreased caspase 3 activation in p53null cells. **(A)** Real-time quantitative PCR (RT-qPCR) of viral RNA detected in IAV-infected A549, A549-KO2, and A549-KO3 cells at 1.5 h post-infection [multiplicity of infection (MOI) = 0.001]. **(B)** Caspase 3/7 activity in mock- or IAV-infected A549 and p53null cells. *p < 0.05. **(C)** Caspase 3/7 activity in mock- or IAV-infected A549 and A549-KO3 cells in the presence (+) or absence (−) of the caspase 3-specific inhibitor Z-DEVD-fmk (40 µM). *p < 0.05. **(D)** Flow cytometry analysis of IAV nucleoprotein (NP) expression in mock- or IAV-infected A549 and A549-KO3 cells, in the presence (+) or absence (−) of the caspase 3-specific inhibitor Z-DEVD-fmk. **(E)** RT-qPCR of viral RNA in the culture supernatant of IAV-infected A549 and A549-KO3 cells, in the presence (+) or absence (−) of the caspase 3-specific inhibitor Z-DEVD-fmk. **(F)** Cellular cytotoxicity in mock- and IAV-infected p53WT and p53null A549 cells. **(G)** Cell viability in mock- and IAV-infected p53WT and p53null A549 cells. For panels **(B–G)**, all measurements were performed at 24 h post-IAV infection (MOI = 0.001).

To determine the extent of initial viral entry into different cells, which may directly affect the viral propagation in a cell population, we simultaneously infected A549 cells, A549-KO2, and A549-KO3 cells and harvested cells at the early time points of 1.5 h post-infection for total RNA extraction. Next, we performed RT-qPCR analysis for viral genes NP and HA. The results showed that the levels of viral RNA are comparable across all cell lines at 1.5 h post-infection, suggesting that at the very early time points, there is no difference for the amount of viral particles entered into different cells and hence, p53 status does not affect initial virus entry (Figure 3A).

### p53-Regulated IAV Susceptibility Is Not Mediated by Caspase 3 Activity

As we observed decreased levels of caspase 3 activity in p53null A549 cells following drug treatment (Figure 1D), we asked whether lower caspase 3 activity in these cells attenuated viral propagation. As seen following the drug treatments, IAV infection increased caspase 3/7 activity in p53WT A549 cells but not in p53null cells (Figure 3B). However, when caspase 3 activation in p53WT A549 cells was specifically inhibited by Z-DEVD-fmk treatment (Figure 3C), the susceptibility of the cells was not significantly altered, as shown by NP labeling *via* flow cytometry (Figure 3D), and by RT-qPCR measuring abundance of the viral genes NP, HA, and NS1 (Figure 3E). Moreover, the difference in caspase 3 activity between p53WT and p53null cell lines did not affect levels of cytotoxicity or viability at 24 h post-infection (Figures 3F,G), which were expectedly low at this time point (10). Taken together, these results indicate that the caspase 3 pathway is not significantly related to the effect of p53 on IAV susceptibility of A459 cells, and that an alternative pathway is responsible.

### Transcriptome Analysis of p53null and p53WT A549 Cells in Response to Influenza Virus Infection

**Figure 4:**
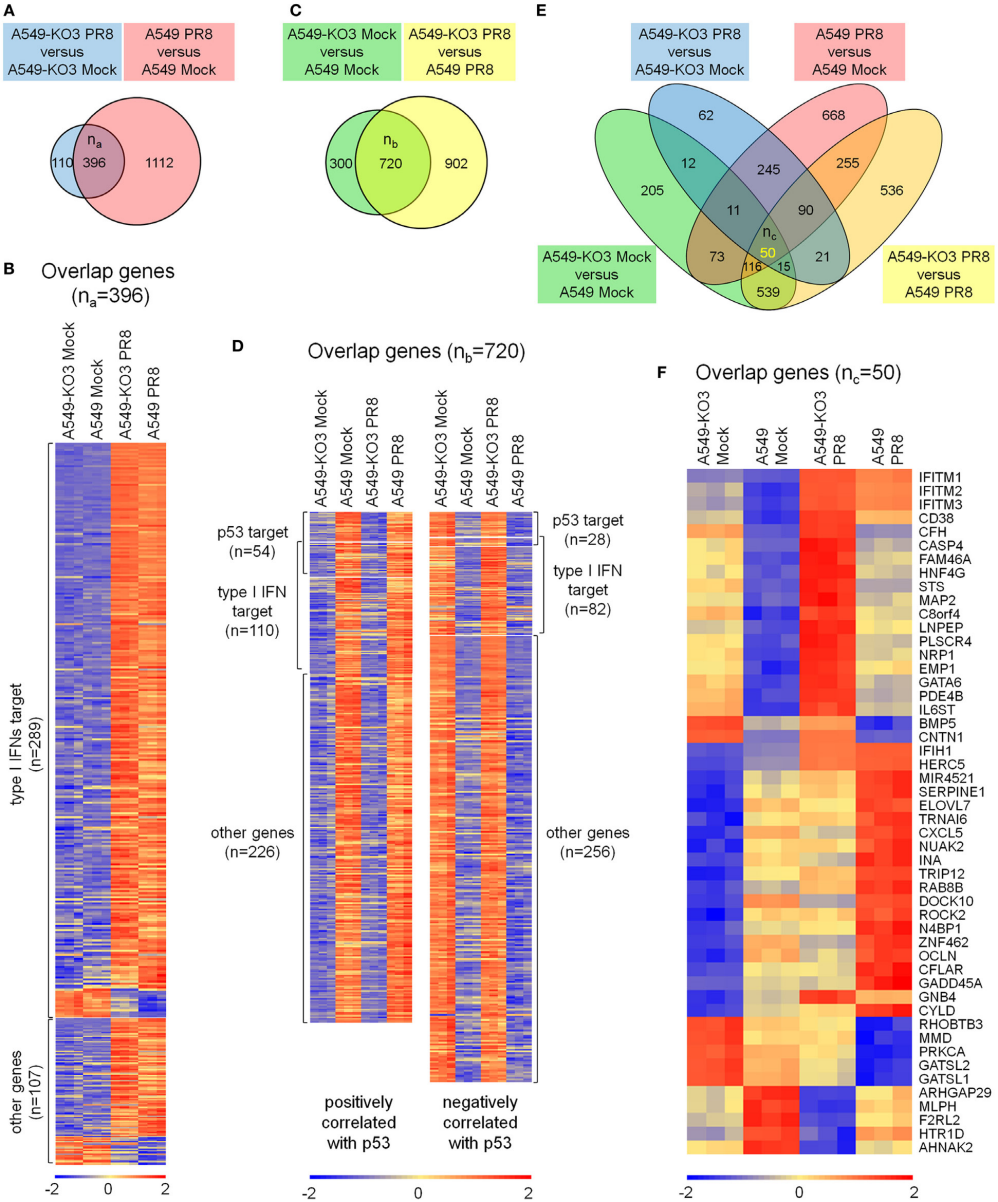
Transcriptome analysis of mock- and IAV-infected A549 and A549-KO3 cells. **(A)** Venn diagram of significantly differentially expressed genes in A549-KO3 PR8 versus A549-KO3 Mock (blue circle) and A549 PR8 versus A549 Mock (red circle). **(B)** Heatmap showing the 396 overlapping genes significantly differentially expressed in both A549-KO3 PR8 versus A549-KO3 Mock, and A549 PR8 versus A549 Mock. **(C)** Venn diagram of significantly differentially expressed genes in A549-KO3 Mock versus A549 Mock (green circle) and A549-KO3 PR8 versus A549 PR8 (yellow circle). **(D)** Heatmap showing the 720 overlapping genes significantly differentially expressed in both A549-KO3 Mock versus A549 Mock, and A549-KO3 PR8 versus A549 PR8. **(E)** Venn diagram of significantly differentially expressed genes in A549-KO3 PR8 versus A549-KO3 Mock (blue), A549 PR8 versus A549 Mock (red), A549-KO3 Mock versus A549 Mock (green), and A549-KO3 PR8 versus A549 PR8 (yellow). **(F)** Heatmap showing the 50 overlapping genes significantly differentially expressed in all four groups.

To understand how p53 and influenza virus replication might be linked, we infected A549 and the representative p53null cell line A549-KO3 with IAV at MOI 0.001 in triplicate, and after 24 h subjected the cells to genome-wide gene expression analysis using the Affymetrix HTA array 2.0 platform. We applied fold change analysis of gene expression to genes within four comparison groups: A549-KO3 PR8 versus A549-KO3 Mock (Group 1); A549 PR8 versus A549 Mock (Group 2); A549-KO3 Mock versus A549 Mock (Group 3); and A549-KO3 PR8 versus A549 PR8 (Group 4) (Table S1 in Supplementary Material). A comparison of the data sets Group 1 and Group 2 revealed 396 overlapping gene features (Figure 4A). The majority of these genes were known type I interferon targets (289/396, 72.9%) (http://www.interferome.org) (18), indicating that IAV infection elicited strong type I interferon responses in both p53WT and p53null cells (Figure 4B; Table S2 in Supplementary Material), as expected (19). Next, we compared data sets Group 3 and Group 4, and the intersection portion contained 720 genes (Figure 4C). Out of the 720 genes, 82 genes have been previously reported as p53 targets (20–22). In addition, 192 genes were known type I interferon targets; interestingly, the expression of 57.3% of these genes was increased by the presence of p53, while levels of expression of the other 82 genes (42.7%) were lower in p53WT A549 cells (Figure 4D; Table S3 in Supplementary Material). We also looked at the expression levels of type I interferons from the transcriptome analysis. Interestingly, all interferon-α genes had not been efficiently induced post-IAV infection, while only the IFNB1 gene encoding the interferon-β1 was sufficiently upregulated. Nevertheless, there is no significant difference between A549 and A549-KO3 cells, either with mock or IAV infection (Figure S3A in Supplementary Material). Following this observation, RT-qPCR analysis was performed to further assess the mRNA expression of IFNB1 gene, which showed similar results as the transcriptome data that IFNB1 mRNA can be induced by IAV infection in both A549 and A549-KO3 cells to a similar extent (Figure S3B in Supplementary Material). When we integrated four datasets, 50 overlapping genes surfaced that had significant fold changes in expression level between all comparison groups (Figure 4E; Table S4 in Supplementary Material). These genes not only responded to influenza virus infection but also to p53 status. The Heatmap of these 50 targets is shown in Figure 4F, and those genes with previously reported associations with the influenza virus life cycle are listed in Table 1.

**Table 1:** p53-regulated genes involved in influenza virus life cycle.

Gene symbol	Expression in infected cells^a^	Expression change (p53null versus p53WT)^b^	Function in viral life cycle	Reference
*IFITM1*	+++	high	Blocks influenza virus infectivity	(3, 7)
*IFITM2*	+++	high	Blocks influenza virus infectivity	(3, 7, 23)
*IFITM3*	+++	high	Blocks influenza virus infectivity	(3, 7, 23)
*FAM46A*	+	high	Blocks influenza virus infectivity	(23)
*IFIH1*	+++	low	Host antiviral defense	(24)
*HERC5*	+++	low	Attenuates viral virulence factor non-structural protein 1	(25)
*SERPINE1*	+	low	Prevents influenza virus maturation	(26)
*ROCK2*	+	low	Permits influenza virus internalization	(27)
*RHOBTB3*	−	low	Assists viral endocytosis	(28)

*^a^+, 0 < log_2_FC < 5; ++, 5 < log_2_FC < 10; +++, log_2_FC > 10; −, log_2_FC < 0*.

*^b^high, higher level in p53null A549-KO3 than p53WT A549 cells; low, lower level in p53null A549-KO3 than p53WT A549 cells*.

Of these genes, the top hits belonged to a family of interferon-regulated antiviral genes—the IFITMs. Expression of IFITMs was higher in p53null cells than in p53WT cells, in both mock- and IAV-infected cell cultures, suggesting that p53 may affect influenza virus infectivity *via* negatively regulating IFITMs. In addition, *FAM46A* was reported to be able to block influenza virus infectivity (23), but its expression in virus-infected cell cultures was relatively low compared with IFITMs. Other genes such as *IFIH1* and *HERC5* were linked to different antiviral effects including attenuating viral virulence factor NS1 (24, 25), which may not be directly associated with the p53-regulated influenza virus propagation pathway. Hence, we hypothesized that p53 modulating the influenza virus infectivity *via* IFITMs was probably a key mechanism in explaining our earlier observations that p53null cells had attenuated IAV propagation.

### p53 Inhibits IFITM Expression During IAV Infection

**Figure 5:**
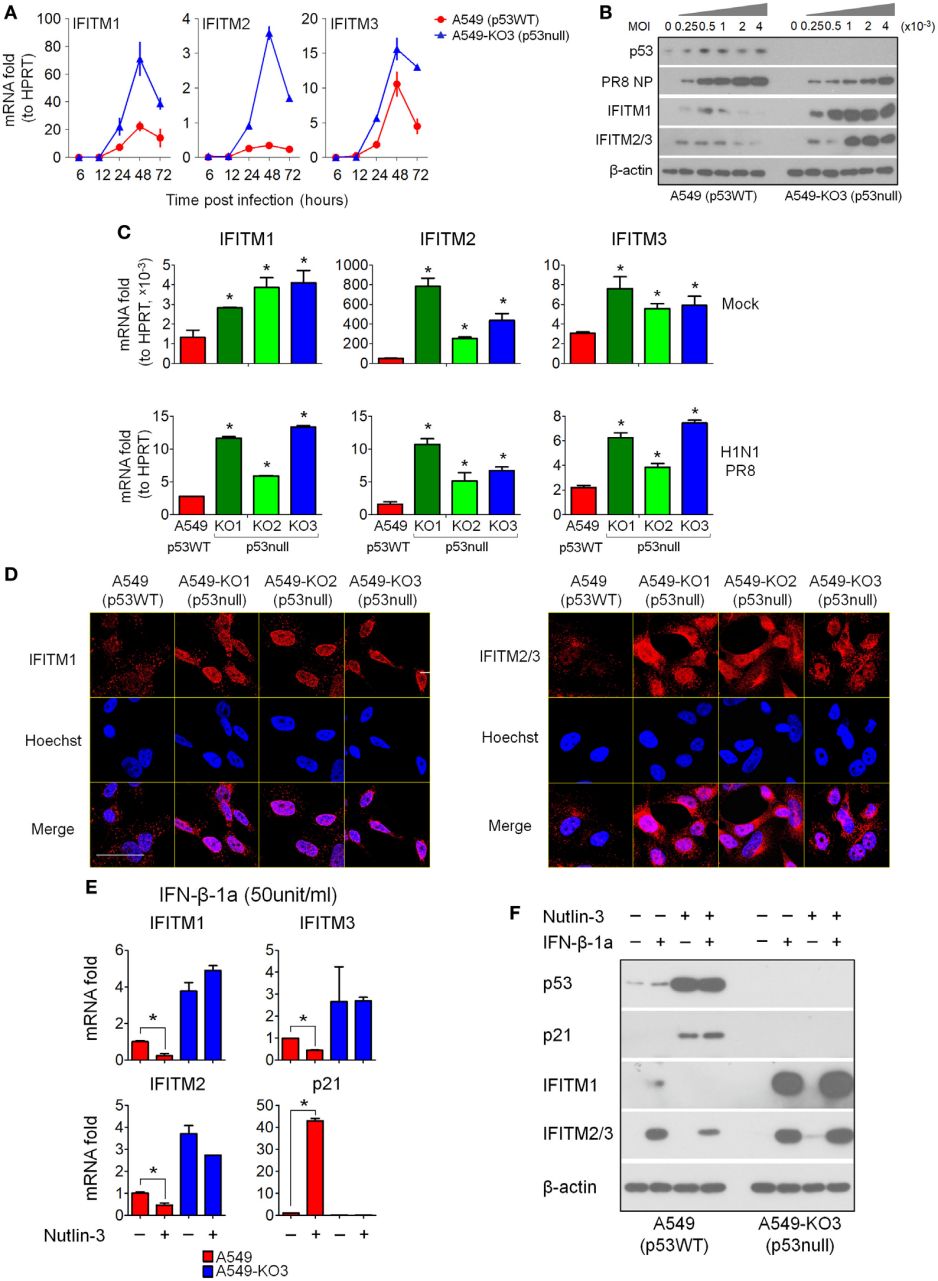
Interferon-induced transmembrane proteins (IFITMs) expression is highly induced by IAV infection and interferons in p53null cells compared with p53WT A549 cells. **(A)** Expression levels of the top 3 most differentially expressed genes (IFITM1, IFITM2, and IFITM3) from the 50 overlapping gene set (Figure 3F) were validated by real-time quantitative PCR (RT-qPCR) validation in IAV-infected A549 and A549-KO3 cells [multiplicity of infection (MOI) = 0.001] at the indicated time points. **(B)** Western blot analysis of expression of viral nucleoprotein (NP), p53, IFITM1, and IFITM2/3 in p53WT A549 and p53null A549-KO3 cells 24 h post-IAV infection at a range of MOI. β-actin blotting was used as a loading control. **(C)** RT-qPCR analysis of IFITM1, IFITM2, and IFITM3 expression level in mock- or IAV-infected p53WT A549, p53null A549-KO1, A549-KO2, and A549-KO3 cells (MOI = 0.001) at 24 h post-infection. *p < 0.05. **(D)** Immunofluorescence microscopy images of IFITM1 and IFITM2/3 expression in IAV-infected p53WT A549, p53null A549-KO1, A549-KO2, and A549-KO3 cells (MOI = 0.001) at 24 h post-infection. Scale bar, 20 µm. **(E)** RT-qPCR analysis of IFITMs mRNA expression level in interferon-β 1a (IFN-β-1a) treated p53WT A549 and p53null A549-KO3 cells pretreated with Nutlin-3 or DMSO solvent control. *p < 0.05. **(F)** Western blot analysis of IFITM1, IFITM2/3, p53, and p21 protein expression level in IFN-β-1a treated p53WT A549 and p53null A549-KO3 cells pretreated with Nutlin-3 or DMSO solvent control. β-actin blotting was used as a loading control. For panels **(E,F)**, both A549 and A549-KO3 cells were treated with Nutlin-3 (25 µM) or DMSO for 6 h, followed by IFN-β-1a treatment (50 U/ml) for another 24 h.

To validate the microarray data, we performed RT-qPCR analysis for RNA expression, and Western blot for protein expression, of IFITM1, IFITM2, and IFITM3. A time-course analysis of IAV-infected A549 and A549-KO3 cells showed that the mRNA expression levels of IFITMs were significantly higher in p53null cells than p53WT cells (Figure 5A). Accordingly, at 24 h post-infection, protein level of IFITMs is markedly higher in A549-KO3 cells across a range of viral MOI, which is associated with lower levels of viral NP, while p53WT cells exhibited only slight increases in IFITM protein levels (Figure 5B). When all p53null cell lines were compared alongside, we saw consistently and significantly higher expression of IFITM mRNAs both at the steady state (mock) and at 24 h post-IAV infection (Figure 5C). We also subjected IAV-infected cells to immunofluorescence confocal microscopy at 24 h post-infection; this revealed that in the absence of p53, IFITM1 is abundant and localized to the perinuclear area and endosomal and lysosomal compartments, while in p53WT A549 cells, there was only weak labeling within endosomes and lysosomes (Figure 5D, left panel). Similarly, IFITM2 and IFITM3 were more abundantly expressed in all three p53null cell lines compared with p53WT A549 cells (Figure 5D, right panel).

Since IFITMs are known to be type I interferon targets, their upregulation during influenza virus infection is likely to result from virus-induced type I interferon signaling. To understand whether p53 was required for interferon-induced IFITM upregulation, we first treated p53WT A549 cells and p53null A549-KO3 cells with recombinant IFN-α1 or IFN-β-1a at two different dosages, and 24 h later, the cells were assayed for IFITM expression by RT-qPCR analysis and Western blot. The results showed that IFITM1, IFITM2, and IFITM3 mRNA expression levels were significantly higher in p53null A549-KO3 cells than p53WT A549 cells, either with or without type I interferon treatment, and independent of the type and dosage of the interferons used (Figure S4A in Supplementary Material). The protein data confirmed the mRNA results, showing much stronger induction of IFITM expression in A549-KO3 cells (Figure S4B in Supplementary Material). Comparable results were seen when we extended our analysis to include A549-KO1 and KO2 cell lines with IFN-β-1a treatment (Figure S5 in Supplementary Material). Finally, we treated A549 and A549-KO3 cells with both IFN-β-1a (50 U/ml) and Nutlin-3 (25 µM), which acts as an Mdm2 antagonist to block p53–Mdm2 interaction and activate p53. We found that pre-treatment of A549 cells with Nutlin-3 further downregulated IFITM1, IFITM2, and IFITM3 expression at both mRNA (Figure 5E) and protein levels (Figure 5F), with accompanying upregulation and accumulation of p53 protein, while in p53null A549-KO3 cells, Nutlin-3 had no effect in modulating the expression of IFITMs.

### Knockdown of IFITMs in p53null Cells Restores IAV Susceptibility

**Figure 6:**
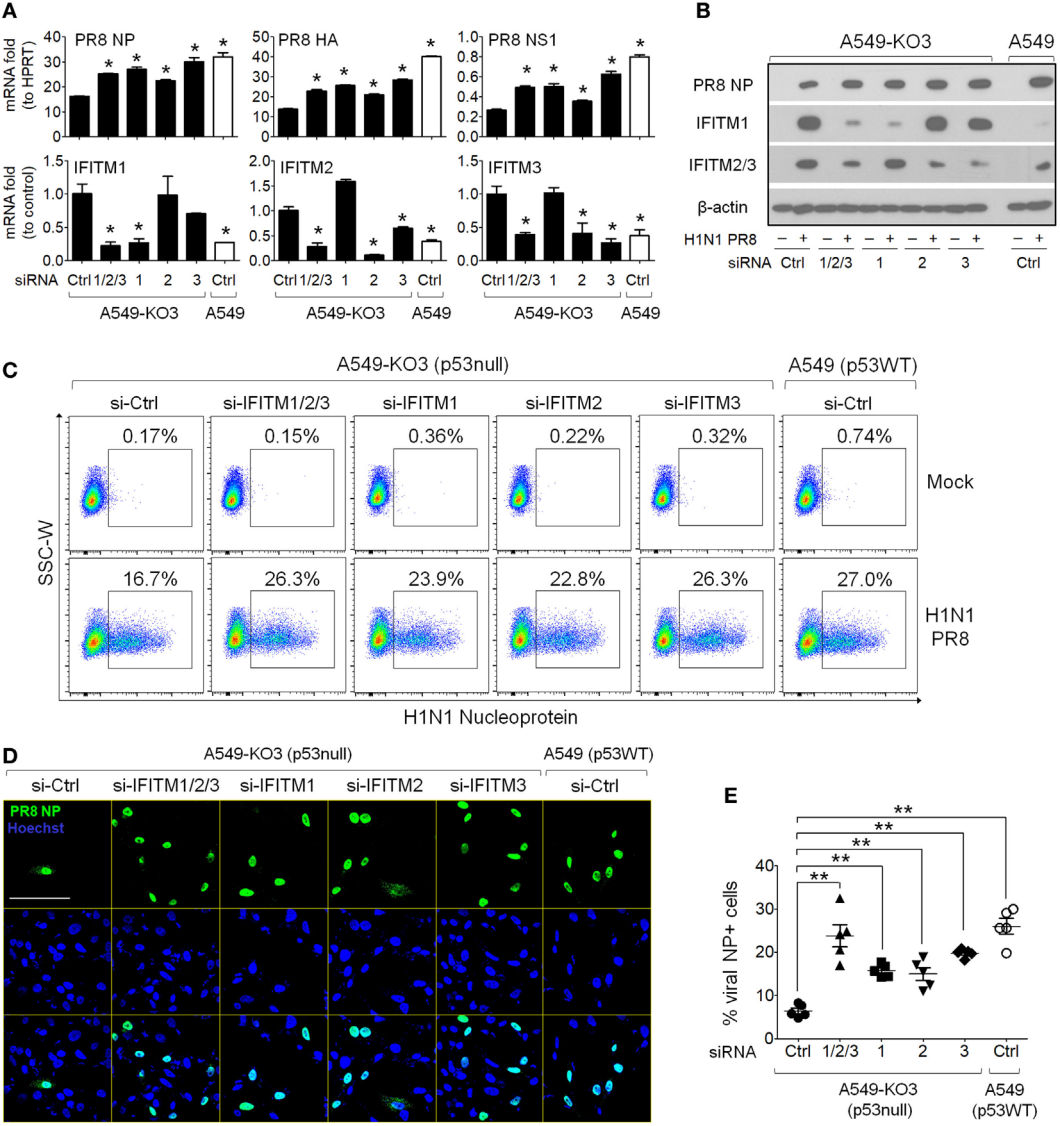
Knockdown of interferon-induced transmembrane proteins (IFITMs) in p53null A549 cells restores susceptibility to IAV infection. A549-KO3 cells were transfected with either IFITM1 (si-IFITM1), IFITM2 (si-IFITM2), IFITM3 (si-IFITM3), a combination of three short interfering RNAs (siRNAs) (si-IFITM1/2/3), or scramble control siRNA (si-Ctrl). 24 h later, cells were detached using trypsin and re-plated onto cell culture plates or μ-slides and after overnight culture, cells were infected with IAV (multiplicity of infection = 0.001) or mock control. 24 h post-infection, cells were harvested for different assays and the RNA and protein lysates were also collected. A549 cells transfected with control siRNA were also included as a benchmark reference. **(A)** Real-time quantitative PCR analysis of IFITM1, IFITM2, IFITM3, IAV nucleoprotein (NP), IAV hemagglutinin (HA), and IAV non-structural protein 1 (NS1) gene expression level in IAV-infected cells pretreated with different siRNAs. For IFITM1, IFITM2, and IFITM3, expression data were normalized to that of the reference gene HPRT, and then to control siRNA transfected A549-KO3 cells, while for NP, HA, and NS1 gene expression, data were normalized to an HPRT control. *p < 0.05. **(B)** Western blot analysis of IAV NP, IFITM1, and IFITM2/3 protein expression in IAV-infected p53WT A549 and p53null A549-KO3 cells pretreated with different siRNAs. β-actin blotting was used as a loading control. **(C)** Flow cytometry analysis of Mock- or IAV-infected p53WT A549 and A549-KO3 cells pretreated with different siRNAs. Each population of NP-positive cells was boxed and their percentages were shown. **(D)** Representative immunofluorescence microscopy images of IAV-infected A549-KO3 cells pretreated with different siRNAs. IAV NP was detected using a FITC-labeled anti-NP antibody, and cell nuclei were stained with Hoechst 33342. Scale bar, 50 µm. **(E)** Percentages of NP-positive cells from IAV-infected A549-KO3 cells pretreated with different siRNAs. Five areas were randomly selected for image capture and the% of NP-positive cells was calculated by the number of NP-positive cells (green) as a proportion of total cell numbers, reflected by Hoechst staining (blue). *p < 0.05.

Interferon-induced transmembrane proteins, especially IFITM3, function as host antiviral factors to block infectivity of a diverse range of viruses, including influenza virus (3, 7, 23). Based on the observations we obtained so far, we hypothesized that the attenuated influenza virus propagation in p53null cell cultures was due to blocked viral infectivity through high levels of IFITM expression induced following viral exposure. To test this hypothesis, we first used siRNAs to knockdown IFITMs in p53null A549-KO3 cells. Commercial pre-designed siRNA pools each targeting IFITM1 (si-IFITM1), IFITM2 (si-IFITM2), and IFITM3 (si-IFITM3) or combined to target all three genes (si-IFITM1/2/3) were transfected into A549-KO3 cells and 48 h later cells were exposed to IAV. At 24 h post-infection, cells were harvested for flow cytometry analysis, fluorescence imaging, and mRNA and protein expression assays. The RT-qPCR results clearly showed that, compared with negative control siRNA (si-Ctrl), si-IFITM1 specifically downregulated IFITM1 expression, while si-IFITM2 and si-IFITM3 downregulated expression of both IFITM2 and IFITM3, possibly due to the high level of sequence homology between these two molecules. Combined use of the three siRNAs (si-IFITM1/2/3) downregulated the expression of all three genes (Figure 6A). These findings were confirmed at the protein level (Figure 6B). When we assessed virus propagation using flow cytometry, A549-KO3 cells pre-transfected with siRNAs to IFITMs had higher frequencies of NP-positive cells compared with control siRNA (si-Ctrl) transfected cells, and the percentage was comparable to the level in control siRNA transfected A549 cells (Figure 6C), suggesting that knockdown of IFITMs in cells lacking p53 restored their IAV susceptibility to p53WT control levels. Immunofluorescence imaging indicated a strong effect of IFITM3 siRNA (si-IFITM3) and combined siRNAs (si-IFITM1/2/3) in particular in mediating increased frequencies of NP-positive cells (Figures 6D,E). Consistently, the production of viral RNA encoding NP, HA, and NS1 was correspondingly upregulated in IFITM-siRNA transfected cells (Figure 6A), as was NP protein expression as visualized by Western blot (Figure 6B).

### Overexpression of IFITMs in p53WT A549 Cells Reduces Their Susceptibility to IAV

**Figure 7:**
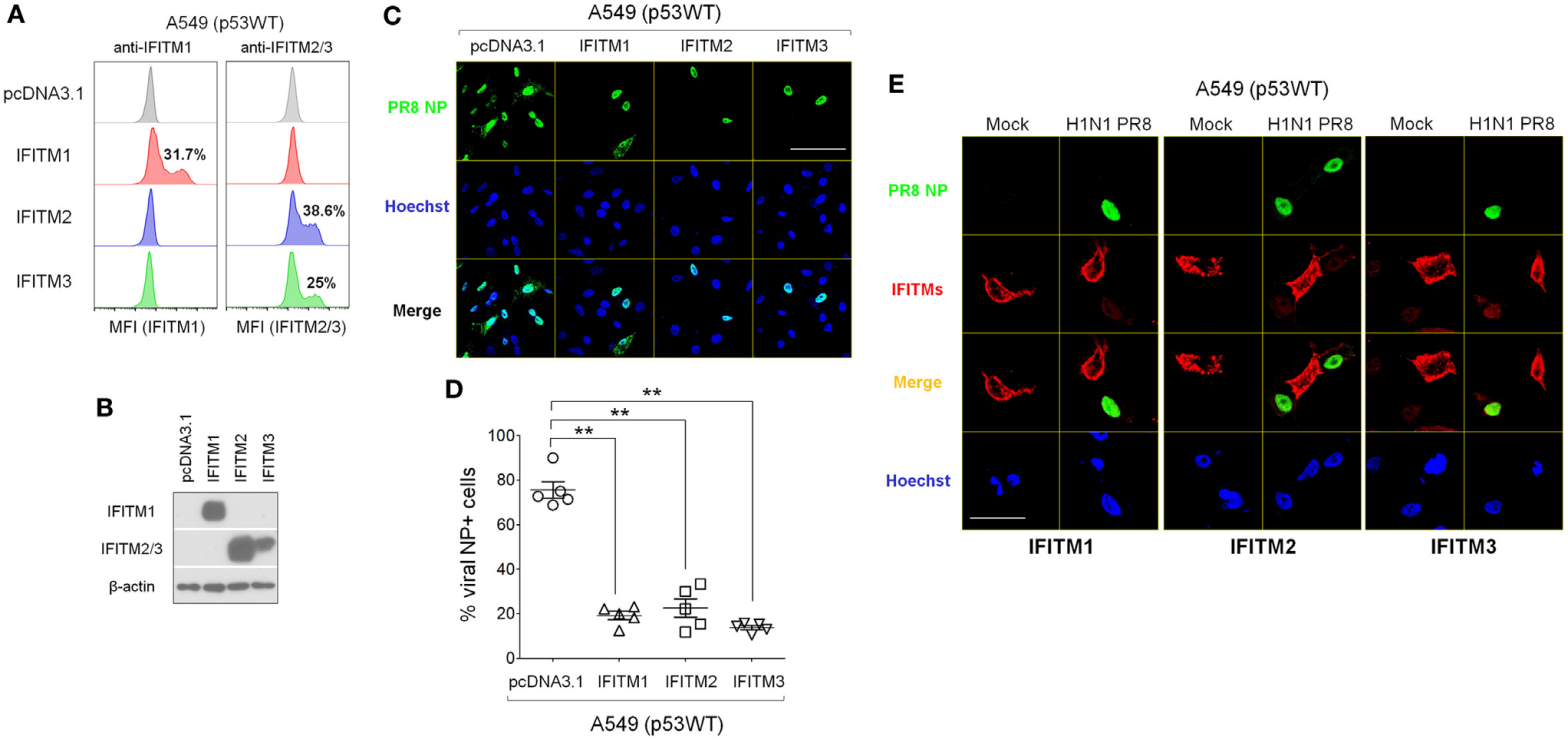
Overexpressed interferon-induced transmembrane proteins (IFITMs) reduce cellular susceptibility to IAV infection. Mammalian expression plasmids encoding human IFITM1, IFITM2, or IFITM3 were constructed and transfected individually into p53WT A549 cells. After 24 h, cells were detached using trypsin and re-plated onto cell culture plates or μ-slides and after overnight culture, cells were infected with IAV (multiplicity of infection = 0.001) or mock control. At 24 h post-infection, cells were harvested for different assays and protein lysates were also collected. **(A)** Transfection efficiency was measured by flow cytometry analysis using IFIMT1- or IFITM2/3-specific antibodies. **(B)** Overexpression of IFITMs in transfected A549 cell cultures was detected by Western blot. **(C)** Representative immunofluorescence microscopy images of IAV-infected A549 cell cultures after transfection with IFITM1, IFITM2, or IFITM3 expression plasmids. IAV nucleoprotein (NP) is shown in green, and Hoechst 33342-stained nuclei in blue. Scale bar, 50 µm. **(D)** Percentage of NP-positive cells in IAV-infected A549 cell cultures that overexpressed IFITM1, IFITM2, or IFITM3 proteins, quantified from fluorescence microscopy images. Five areas were randomly selected for image capture and the percentages of NP-positive cells (green) were calculated relative to the total cell numbers, reflected by Hoechst staining (blue). **p < 0.01. **(E)** Immunofluorescence microscopy images of IAV-infected A549 cells transfected with IFITM1, IFITM2, or IFITM3 expression plasmids. IFITMs are shown in red, IAV NP in green, and Hoechst 33342-stained nuclei in blue. Scale bar: 20 µm.

Next, we investigated how increasing the expression of IFITMs in p53WT A549 cells, which normally does not efficiently increase IFITM expression during IAV infection (Figure 5B), affected their susceptibility to IAV. IFITM mRNA was extracted and expanded by reverse transcriptase PCR using cDNA derived from IAV-infected A549 cells. The PCR products were purified and cloned into the pcDNA3.1 (+) plasmid and the plasmid constructs encoding IFITM1, IFITM2, and IFITM3 were separately transfected into A549 cells. After 48 h, all three constructs had induced elevated levels of their respective IFITM protein expression in transfected cells (Figures 7A,B), which was associated with significantly lower frequencies of NP-positive cells compared with empty vector control-transfected A549 cells (Figures 7C,D; Figure S6 in Supplementary Material). Immunofluorescence imaging indicated that viral NP expression was restricted to those cells within the culture that were not over-expressing IFITM1, IFITM2, or IFITM3 (Figure 7E).

### p53 Regulates IFITM Expression Independent of Its Transcriptional Activity

**Figure 8:**
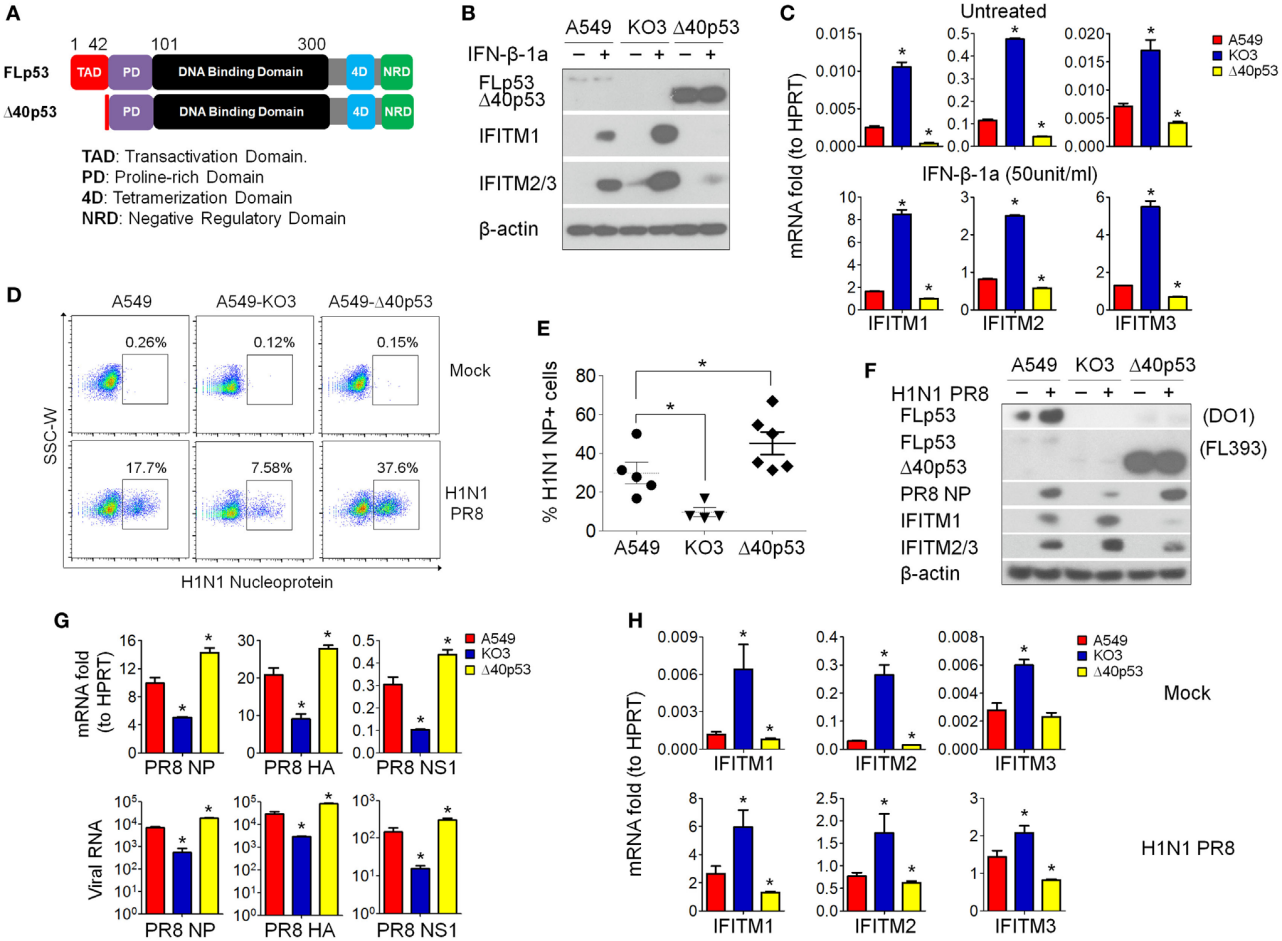
p53 regulates interferon-induced transmembrane proteins (IFITMs) independent of its transcriptional activity. **(A)** Schematic presentation of modular structures of full-length p53 and short isoform Δ40p53 proteins. **(B)** Western blot analysis of p53 (detected by antibody FL393, sc-6243), IFITM1, and IFITM2/3 expression levels in interferon-β 1a (IFN-β-1a) treated p53WT A549, A549-KO3, and A549-Δ40 cells. β-actin blotting was used as a loading control. **(C)** Real-time quantitative PCR (RT-qPCR) analysis of IFITMs mRNA expression in IFN-β-1a treated p53WT A549, A549-KO3, and A549-Δ40 cells. *p < 0.05. **(D)** A549, A549-KO3, and A549-Δ40 were infected with IAV [multiplicity of infection (MOI) = 0.001] and after 24 h were analyzed for IAV nucleoprotein (NP) expression by flow cytometry. The populations of NP-positive cells were boxed and their percentages shown. **(E)** Percentage of NP-positive cells in IAV- (MOI = 0.001) -infected p53WT A549, A549-KO3, and A549-Δ40 cells quantified from fluorescence microscopy images: between 4 and 6 areas were randomly selected for image capture and the percentages of NP-positive cells within the total cell population were calculated. *p < 0.05. **(F)** Western blot analysis of p53 (detected by both DO-1, sc-126 and FL393, sc-6243), IAV NP, IFITM1, and IFITM2/3 protein expression in p53WT A549, A549-KO3, and A549-Δ40 cells following IAV infection (MOI = 0.001). β-actin blotting was used as a loading control. **(G)** RT-qPCR measurement of viral RNAs encoding NP, hemagglutinin (HA), and non-structural protein 1 (NS1) genes detected either in IAV- (MOI = 0.001) infected cells (top) or from the culture supernatant (bottom). *p < 0.05. **(H)** RT-qPCR analysis of IFITM1, IFITM2, and IFITM3 gene expression in mock- or IAV-infected p53WT A549, A549-KO3, and A549-Δ40 cells. *p < 0.05.

As IFITMs were identified in the comparative gene expression screening as differentially expressed during infection and dependent on p53 status (Figure 4F; Table 1), we next asked whether this effect was directly mediated by p53’s transcriptional activity, or *via* indirect mechanisms. Using a sgRNA construct that specifically targets the second exon of p53, we generated isogenic A549 cells (designated as “A549-Δ40p53”) in which the full-length p53 protein was destroyed but sparing a naturally occurring p53 short isoform Δ40p53. Δ40p53 lacks the first 39 amino acids of full-length p53 and thus lacks transcriptional regulation activity (29) (Figure 8A). A549-Δ40p53 exhibited an accumulation of Δ40p53 (Figure 8B), as expected, due to lack of negative feedback regulation of Mdm2, which otherwise would be transcriptionally induced by intact full-length p53 and functions in p53 ubiquitination and degradation (30, 31). We then treated A549-Δ40p53 cells with IFN-β-1a and found a profound inhibition of IFITM upregulation at both the protein (Figure 8B) and mRNA level (Figure 8C). Thus, differential IFITM expression in these cells is indeed regulated at the transcriptional level, but *via* a transcriptional mediator that interacts, either directly or indirectly, with both the long and short isoforms of p53.

The above data suggested that the amount of cellular p53 (full-length or short isoform) was correlated with the extent of inhibition of IFITM expression following interferon treatment of A549 cells. We therefore hypothesized that the A549-Δ40p53 cells would also less efficiently upregulate IFITM expression in response to IAV infection, and so would be highly susceptible to the virus. Indeed, when we simultaneously infected p53WT A549, p53null A549-KO3, and A549-Δ40p53 cells with IAV, A549-Δ40p53 cells showed the highest percentage of viral NP-positive cells (37.6%) as measured by flow cytometry (Figure 8D), fluorescence imaging (Figure 8E; Figure S7 in Supplementary Material), and Western blot (Figure 8F). We also compared levels of viral RNAs encoding NP, HA, and NS1 in cell lysates (Figure 8G, top panel) and culture supernatants from A549, A549-Δ40p53, and A549-KO3 cells (Figure 8G, bottom panel), and saw highest levels of all viral genes in A549-Δ40p53 cells. Accordingly, the transcript levels of IFITMs exhibited the opposite pattern, being lowest in A549-Δ40p53 under both mock and IAV infection conditions (Figure 8H).

**Figure 9:**
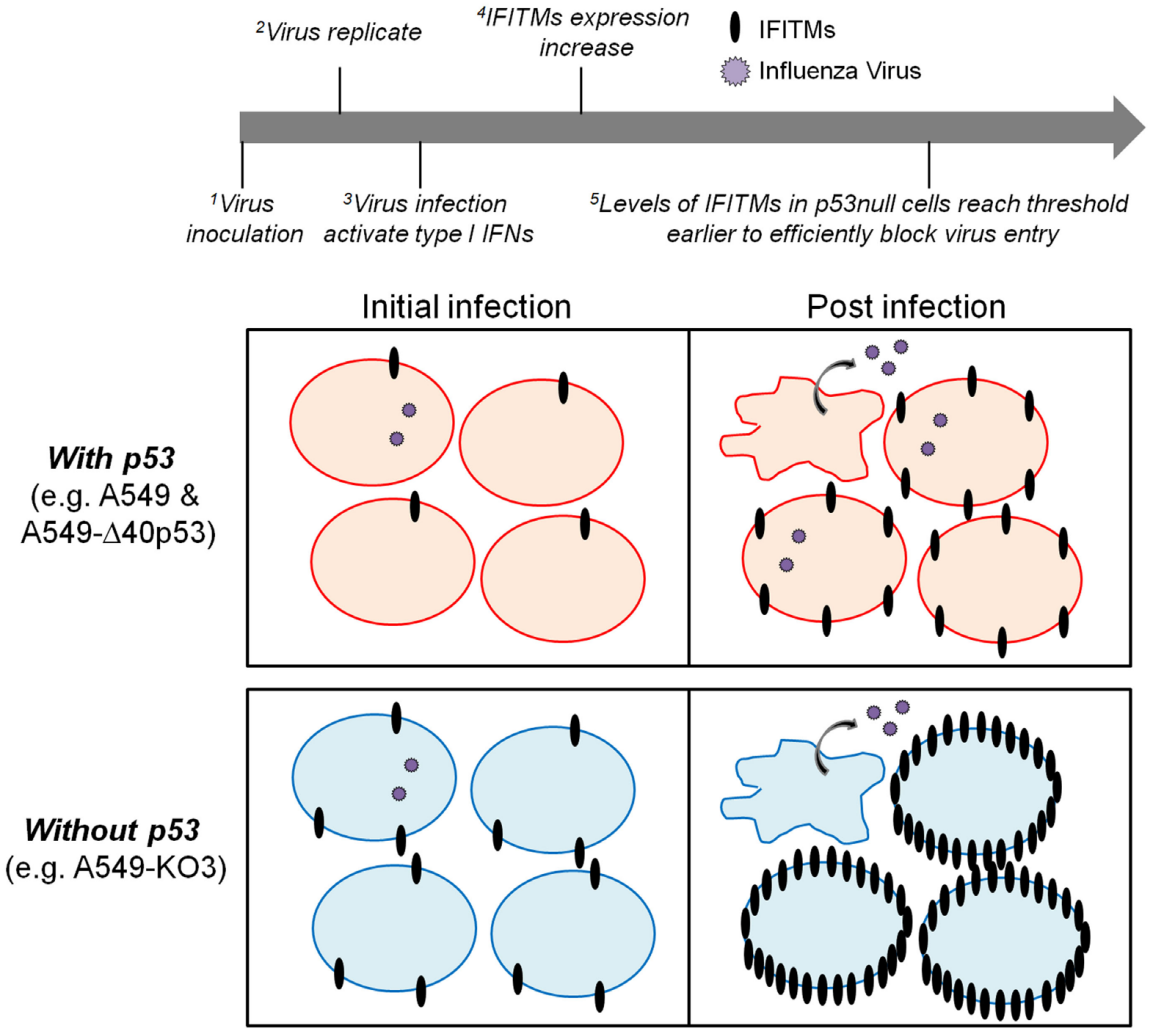
A proposed model of p53 and interferon-induced transmembrane protein (IFITM) cross-regulation of cellular IAV susceptibility. During the initial influenza virus inoculation (initial infection), both p53-sufficient (top left panel) and -deficient cells (bottom left panel) express extremely low levels of IFITMs and hence are equally susceptible to virus infection. With the progression of virus replication (post-infection), the type I interferon pathways are activated to increase the expression of IFITMs. In cells expressing p53, IFITMs could not be efficiently upregulated so that the viral particles are still able to infect the neighboring cells (top right panel). While in cells without p53 expression, IFITMs could be more efficiently upregulated so that the threshold will be reached earlier to block the viral infectivity successfully (bottom right panel).

Taken together, the above data suggest a novel model of the role of p53 in determining cellular IAV susceptibility: during initial IAV exposure, both p53-sufficient and -deficient A549 cells express very low levels of IFITMs and are equally susceptible to incoming virus (Figure 3A). However, as IAV infection progresses and stimulates cell stress and type I interferon pathways, p53 expressing cells are unable to effectively upregulate expression of protective IFITMs and remain susceptible to high levels of IAV infection and propagation (Figure 9). While IFITM expression is inhibited at the transcriptional level, this is not mediated by p53 directly, but rather by an as-yet-unidentified downstream mediator with gene regulatory capacity.

## Discussion

In this study, we exploited CRISPR/Cas9 genome-editing technology to generate p53null cell lines from a lung carcinoma cell line A549 and to explore their susceptibility to IAV infection. Using these isogenic p53null cells, we clearly showed that the level of p53 positively correlates with viral load in cultures infected with influenza A virus H1N1 PR8 strain. We went on to demonstrate that wild-type p53 indirectly and negatively regulates expression of the interferon-inducible antiviral protein family members IFITM1, IFITM2, and IFITM3 during IAV infection, and that manipulating the levels of IFITM in cells can rescue their susceptibility driven by p53 expression. This previously unrecognized association between human p53 and the broad spectrum antiviral IFITM protein led us to propose a novel model of IAV susceptibility in A549 cells.

While many viruses actively block p53 (9), its activation is a hallmark of influenza virus infection (10). Induction and activation of p53 in this setting serves the host by inducing apoptosis of infected cells (10), and enhancing cellular innate immune responses, including antiviral interferon pathways (32). Similarly, studies in mice have shown that animals lacking p53 have higher viral loads and exacerbated disease during IAV infection, compared with wild-type controls (11). However, it seems that IAV may have evolved to exploit p53 to benefit its own life cycle: expression of p53 was shown to be positively associated with higher viral protein level in A549 cells (12, 14). These apparently contradictory conclusions may stem in part from differences in the models and experimental settings used; however, until now it has not been clear how p53 affects IAV infection in potentially susceptible cell populations.

Using isogenic p53WT and p53null cells generated from the human cell line A549 for parallel analysis, we have identified the IFITMs family of genes which are known to reduce the cellular susceptibility to IAV infection as downstream targets of the p53 pathway. In agreement with our data, Daniel-Carmi et al. previously showed that the steady-state level of IFITM2 in HCT116 cells was downregulated by p53 expression (33); while in mice, in contrast, recent genome-wide screening showed that IFITM1 expression was significantly lower in IAV-infected p53 knockout animals, which were more susceptible to the virus compared with wild-type mice (34). These data urge caution in translating findings from the murine system into human IAV infections and may also explain the findings of Munoz-Fontela et al., who concluded that p53 was protective against IAV infection in mice (11). Nevertheless, it would still be interesting to test this p53 regulation of IFITMs and IAV infectivity in other relevant animal models such as in pig (35), with the recent success of generating p53 biallelic knockout *Diannan* miniature pigs (36).

Having identified IFITMs as downstream targets of p53 during IAV infection by gene expression analysis, we then asked whether p53 was directly regulating their transcription, or whether the interaction required additional molecular partners. First, to test if the p53 regulation on IFITMs in response to IAV infection is due to the differences between p53WT and p53null cells in inducing the expression of type I interferons, we assessed the mRNA expression of interferons by the transcriptome data and RT-qPCR analysis (Figure S3 in Supplementary Material). Both measurements suggest that p53 regulates IFITMs independent of the levels of type I interferons as there are no significant difference between the A549 and A549-KO3 cells in inducing interferons post-IAV infection. Second, we generated an isogenic A549 cell line that expressed and accumulated only the short p53 isoform, Δ40p53, which lacks transcriptional activity (29), and found a higher level of IFITM inhibition (1.5–6 times higher than A549 cells, Figures 8C,H), accompanied by elevated IAV susceptibility. This finding not only further supports our hypothesis that p53 regulates influenza virus infectivity *via* modulating the levels of IFITMs but also shows that such regulation occurs independent of p53’s transcriptional activity. In their study on IFITM2 and p53, Daniel-Carmi et al. suggested that p21, a downstream transcriptional target of p53, might mediate the repression of IFITM2 in HCT116 cells (33); however, our data exclude this possibility in the A549/IAV system. Nevertheless, as a well-known p53 transcriptional target and a regulator of multiple protein kinases, p21 may also impact on IAV infection. For example, Pascua et al. reported that p21-activated kinases enhanced the viral titer in A549 cells during IAV infection (strain H1N1/A/WSN/1933) (37).

Interestingly, a more recent study by Nailwal et al. (38) revealed that IAV NP protein induces attenuation of host ubiquitin ligase RNF43 to stabilize the cellular p53, and depletion of RNF43 enhanced viral replication. In this study, a much higher MOI (MOI = 5) was applied which allows almost all cultured cells to be infected at the initial inoculation, and hence only viral replication rather than viral infectivity could be investigated. While in our current study, a much lower MOI of 0.001 was applied to better mimic the physiological conditions of infection so that the viral infectivity in a cell population could be assessed. Another compelling report by Terrier et al. (39) showed that IAV infection control the expression of two proviral human p53 isoforms p53β and Δ133p53α. While Terrier’s study aimed to examine the differential role of the different p53 isoforms on IAV replication by siRNA knockdown to remove one specific isoform at a time, our current study, using CRISPR/Cas9-mediated gene knockout to globally knockout all p53 isoforms, focused on how a complete p53 null phenotype affects the IAV infectivity. Furthermore, Turpin et al. suggested that p53 expression decreased IAV replication in A549 cells, based on experiments using a transcriptionally inactive p53 mutant designed to create a functional p53 knockout (10): these data align with our findings in A549-Δ40p53 cells, which similarly showed increased IAV susceptibility. It will be interesting in future studies to further characterize the specific domains of p53 necessary for indirect IFITM regulation to understand how the conclusions of these studies might be fully reconciled.

In conclusion, our study provides the evidence that the level of human p53 protein negatively correlates with the expression level of antiviral family proteins IFITM1, IFITM2, and IFITM3, which in turn determines the susceptibility of A549 cells to IAV. These data propose a novel mechanism by which influenza virus may harness host p53 to restrict IFITM upregulation and thereby extend the early window of susceptibility of its target cell population to favor viral infectivity.

## Author Contributions

BW and ER conceived this project. BW designed all experiments and analyzed the data. BW, MS, and ZY conducted the experiments. TL and JC analyzed the Exon array data. BW and ER wrote and revised the manuscript.

## Conflict of Interest Statement

The authors declare that the research was conducted in the absence of any commercial or financial relationships that could be construed as a potential conflict of interest.
