# Supplementary material for: Influenza A Virus Facilitates Its Infectivity by Activating p53 to Inhibit the Expression of Interferon-Induced Transmembrane Proteins
Source: Front Immunol. 2018 May 31;9:1193. doi: 10.3389/fimmu.2018.01193 (PMC5990591; doi:10.3389/fimmu.2018.01193)
Supplement: Supplementary file 5 [file data_sheet_1.docx]

**Supplementary Material**

**Influenza virus facilitates its infectivity**

**by activating p53 to inhibit the expression of**

**interferon-induced transmembrane proteins (IFITMs)**

**Supplementary Materials and Methods**

The primers used for real-time qPCR analysis in the current study are shown below:

*PR8 NP* forward: CATCAAAGGGACGAAGGTGC

*PR8 NP* reverse: ACTTCTGGTCCTTATGGCCC

*PR8 HA* forward: TTGCTAAAACCCGGAGACAC

*PR8 HA* reverse: CCTGACGTATTTTGGGCACT

*PR8 NS1* forward: CAGCACTCTTGGTCTGGACA

*PR8 NS1* reverse: TGGACCATTCCCTTGACATT

*IFITM1* forward: TGCACAAGGAGGAACATGAG

*IFITM2* forward: GAGCAGGAAGTGGCTATGCT

*IFITM3* forward: CTCAAGGAGGAGCACGAGGT

*IFITM1*, *IFITM2*, *IFITM3* reverse: CTTCCTGTCCCTAGACTTCACG

*CDKN1A*/p21 forward: GACACCACTGGAGGGTGACT

*CDKN1A*/p21 reverse: GGATTAGGGCTTCCTCTTGG

*IFNB1* forward: GCCTCAAGGACAGGATGAAC

*IFNB1* reverse: AGCCAGGAGGTTCTCAACAA

*HPRT* forward: GTAATGACCAGTCAACAGGGGAC

*HPRT* reverse: CCAGCAAGCTTGCGACCTTGACCA

The primers used for IFITMs cloning in the current study are shown below:

IFITM1-CDS-F: ATGCACAAGGAGGAACATGA (1^st^ PCR)

IFITM1-CDS-R: CTAGTAACCCCGTTTTTCCTGTAT (1^st^ PCR)

IFITM1-IF-F1: TCGGATCCATGCACAAGGAGG (2^nd^ PCR)

IFITM1-IF-R1: GACTCGAGCTAGTAACCCCGTT (2^nd^ PCR)

IFITM1-IF-F2: TACCGAGCTCGGATCCATGCA (3^rd^ PCR)

IFITM1-IF-R2: GCCCTCTAGACTCGAGCTAGTAACC (3^rd^ PCR)

IFITM2-CDS-F: ATGAACCACATTGTGCAAACCTTCTCT (1^st^ PCR)

IFITM2-CDS-R: CTATCGCTGGGCCTGGACGA (1^st^ PCR)

IFITM2-IF-F1: GCTCGGATCCATGAACCACATTGTG (2^nd^ PCR)

IFITM2-IF-R1: CTCGAGCTATCGCTGGGCCT (2^nd^ PCR)

IFITM2-IF-F2: TACCGAGCTCGGATCCATGAACCA (3^rd^ PCR)

IFITM2-IF-R2: GCCCTCTAGACTCGAGCTATCGCT (3^rd^ PCR)

IFITM3-CDS-F: ATGAATCACACTGTCCAAACCTT (1^st^ PCR)

IFITM3-CDS-R: CTATCCATAGGCCTGGAAGATCA (1^st^ PCR)

IFITM3-IF-F1: TCGGATCCATGAATCACACTGTCC (2^nd^ PCR)

IFITM3-IF-R1: GACTCGAGCTATCCATAGGCCT (2^nd^ PCR)

IFITM3-IF-F2: TACCGAGCTCGGATCCATGAATCA (3^rd^ PCR)

IFITM3-IF-R2: GCCCTCTAGACTCGAGCTATCCATA (3^rd^ PCR)

**Supplementary Tables**

**Table S1** Fold change in gene expression of all differentially expressed genes within four comparison groups. Tab 1: A549-KO3 PR8 versus A549-KO3 Mock (Group1); Tab2: A549 PR8 versus A549 Mock (Group 2); Tab 3: A549-KO3 Mock versus A549 Mock (Group 3); and Tab 4: A549-KO3 PR8 versus A549 PR8 (Group 4).

**Table S2** Normalized gene expression of 396 overlapping genes among Group 1 (A549-KO3 PR8 versus A549-KO3 Mock) and Group 2 (A549 PR8 versus A549 Mock).

**Table S3** Normalized gene expression of 720 overlapping genes among Group 3 (A549-KO3 Mock versus A549 Mock) and Group 4 (A549-KO3 PR8 versus A549 PR8).

**Table S4** Normalized gene expression of 50 overlapping genes among all four comparison groups.

**Supplementary Figures**

**
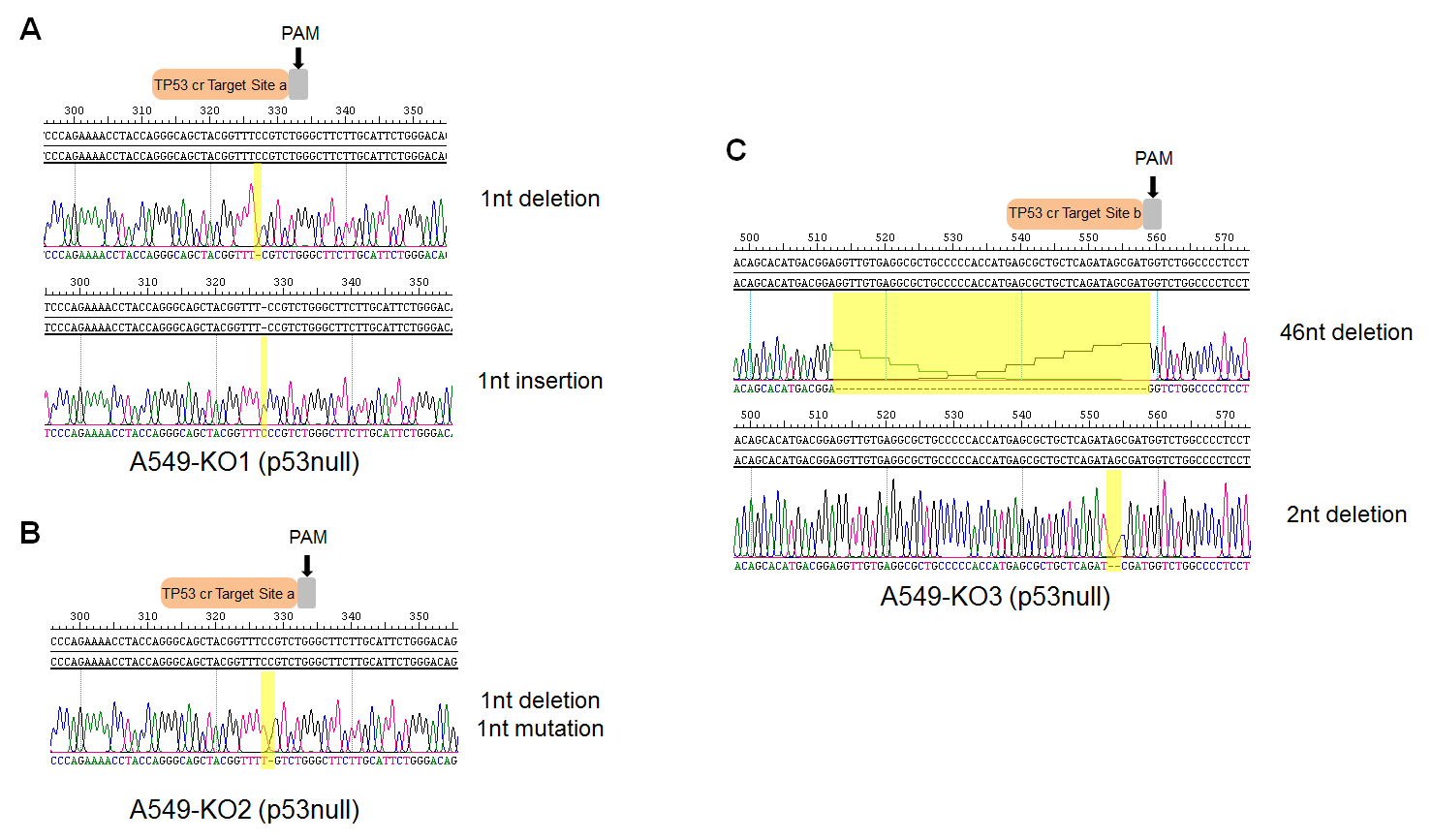
**

**Figure S1** Sequencing analysis of p53null A549 cell clones generated by CRISPR/Cas9 technology. DNA modifications of different p53null cell clones A549-KO1 **(A)**, A549-KO2 **(B)** and A549-KO3 cells **(C)**.


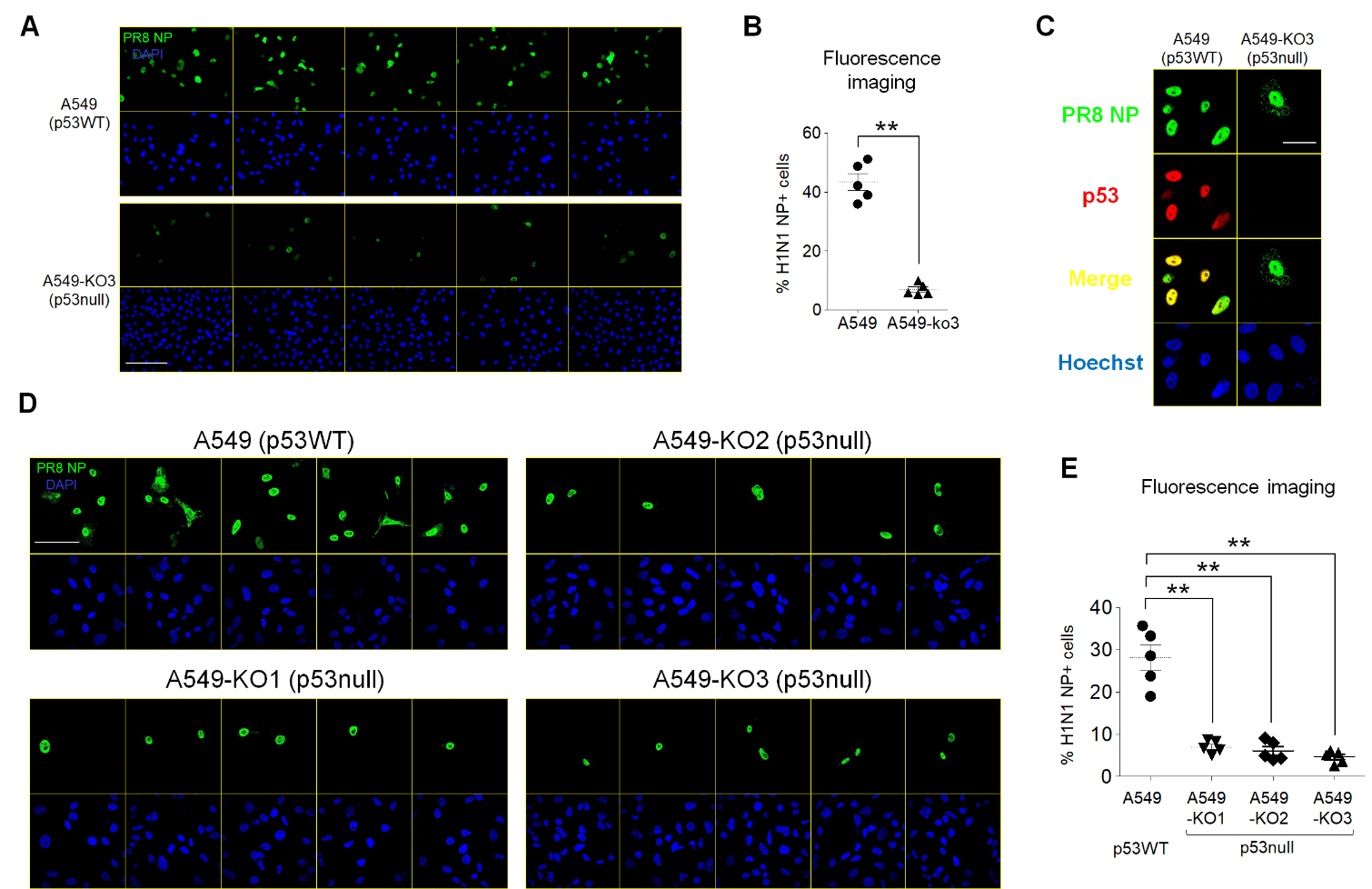


**Figure S2** CRISPR/Cas9-generated p53null A549 cells were less susceptible to IAV infection compared to p53WT A549 cells. **(A)** Immunofluorescence microscopy images of p53WT A549 and A549-KO3 cells at 24 hours post-IAV infection (MOI=0.001). IAV NP protein is shown in green, with Hoechst 33342-stained nuclei in blue. Five areas were randomly-selected for image capture. Scale bar, 100µm. **(B)** Percentage of NP-positive cells in IAV-infected p53WT A549 and A549-KO3 cultures, expressed relative to the total number of cells. **(C)** Confocal microscopy imaging of p53WT A549 and A549-KO3 cells, showing IAV NP in green and cellular p53 in red. Scale bar, 20µm. **(D)** Immunofluorescence microscopy images of IAV-infected p53WT A549, A549-KO1, A549-KO2 and A549-KO3 cells at 24 hours post infection (MOI=0.001). IAV NP is shown in green, with Hoechst 33342-stained nuclei in blue. Five areas were randomly selected for image capture. Scale bar, 50µm. **(E)** Percentage of NP-positive cells in IAV-infected p53WT A549, A549-KO1, A549-KO2 and A549-KO3 cultures is expressed relative to the total number of cells. ***p*<0.01.


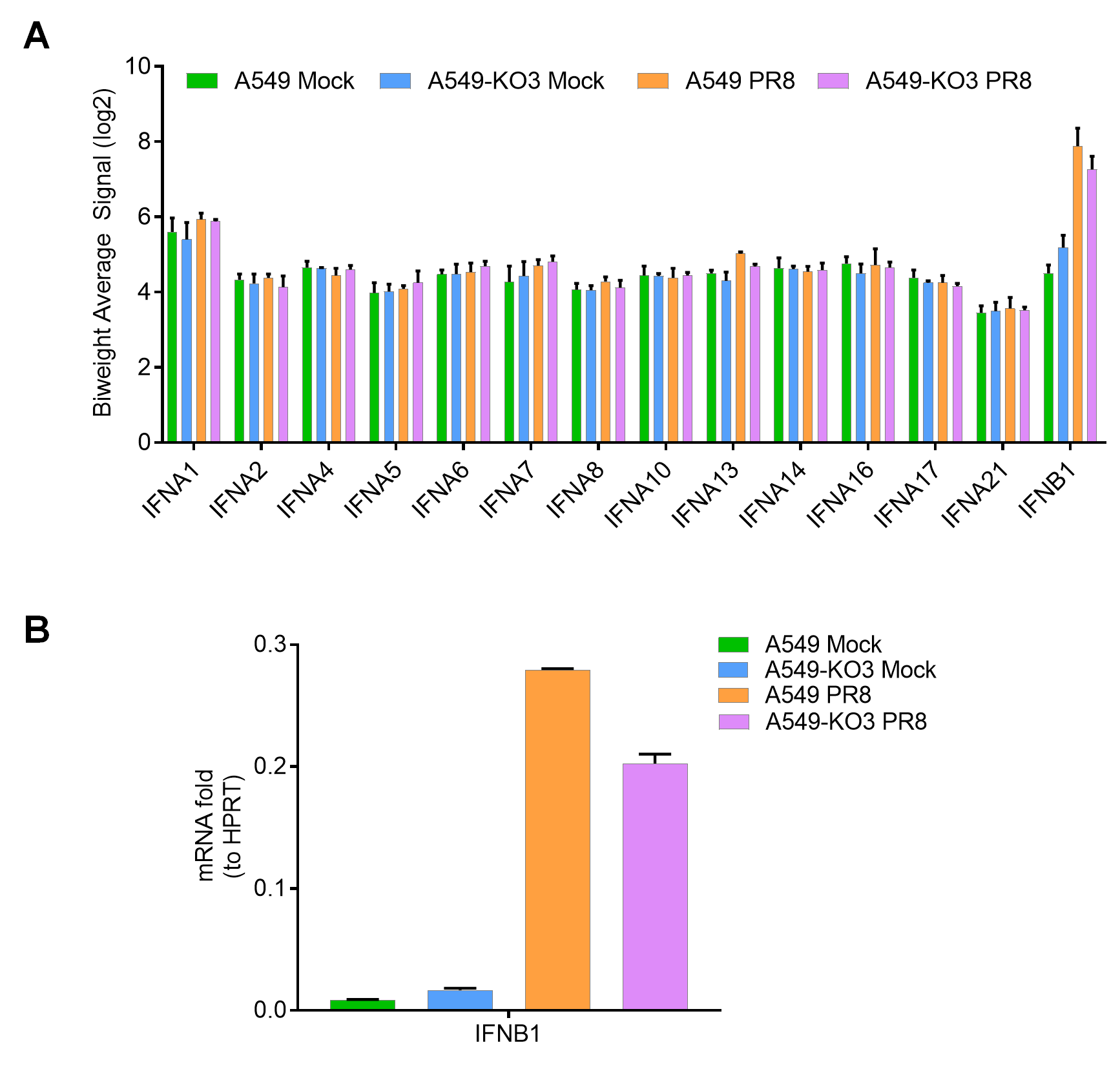


**Figure S3** Expression of type I interferon genes in mock and IAV infected A549 and A549-KO3 cells.

**(A)** Biweight average signals (log2) of each interferon-alpha genes and interferon-beta 1 from Affymetrix ExonArray data. **(B)** RT-qPCR analysis of IFNB1 gene expression in A549 and A549-KO3 cells in response to IAV PR8 infection at 24 hours post infection (MOI = 0.001).

**
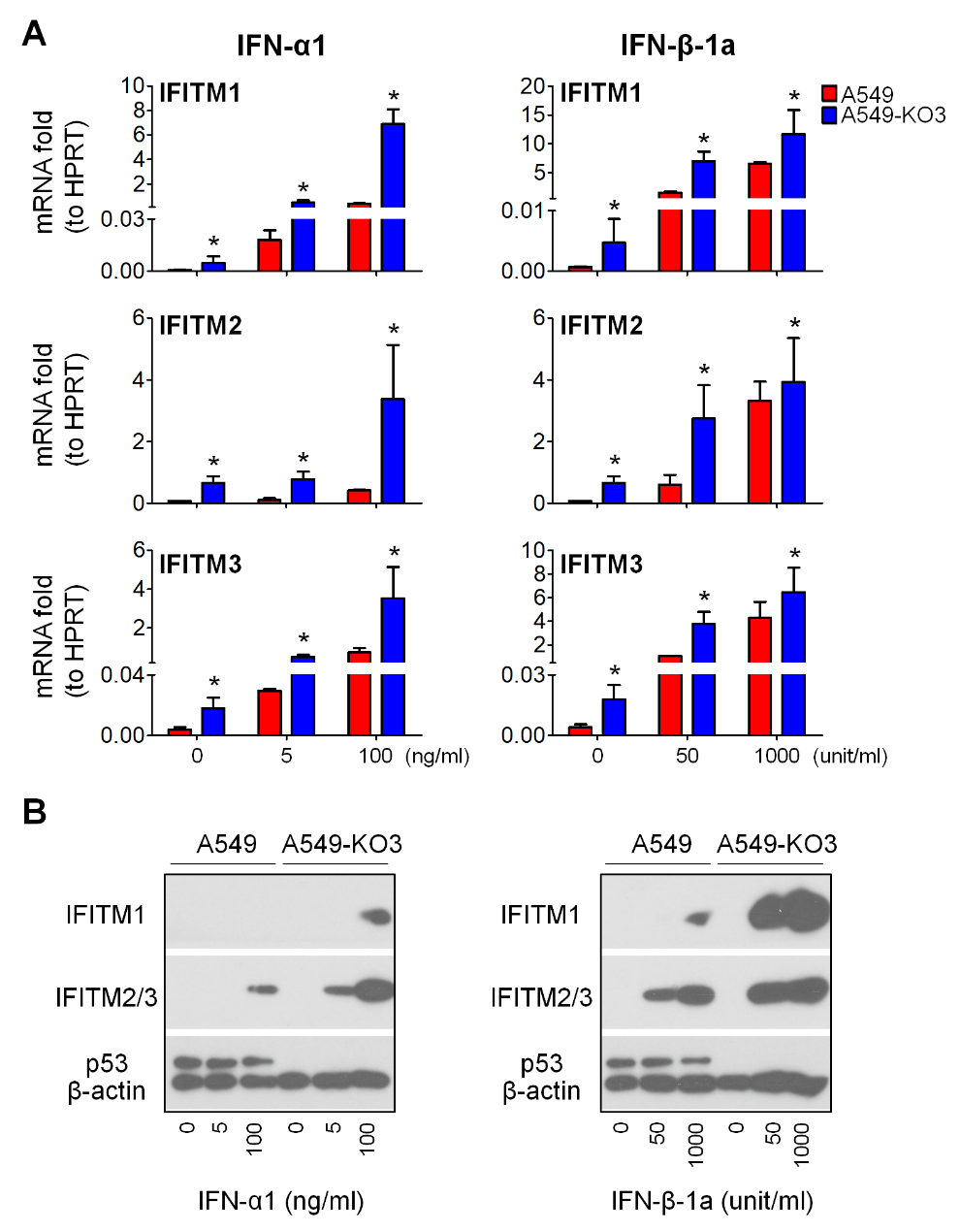
**

**Figure S4** IFITM expression is more highly up-regulated in response to type I interferons in A549-KO3 cells compared to A549 cells. **(A)** Real-time qPCR analysis of IFITM1, IFITM2, and IFITM3 gene expression in IFN-α1 (5 or 100ng/ml) and IFN-β-1a (50 or 1000unit/ml) treated p53WT A549 cells and A549-KO3 cells at 24 hours post treatment. **(B)** Western blot analysis of IFITM1, IFITM2, and IFITM3 protein expression in IFN-α1 (5 or 100ng/l) and IFN-β-1a (50 or 1000 unit/ml) treated p53WT A549 cells and p53null A549-KO3 cells at 24 hours post treatment. **p*<0.05. *p* values were calculated on the significance of differences between A549-KO3 and control A549 cells.


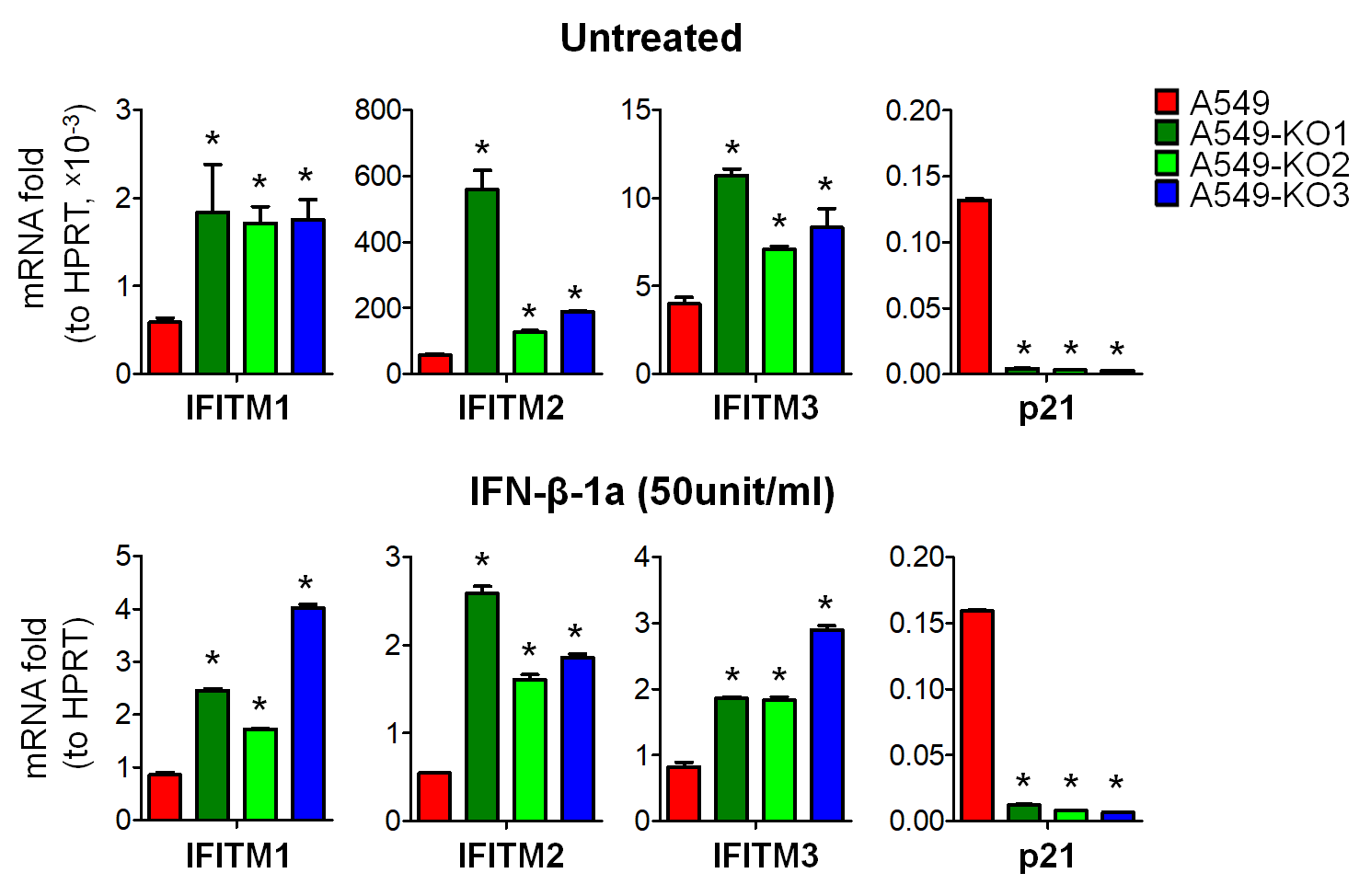


**Figure S5** IFITM mRNAs are more highly expressed and up-regulated in response to IFN-β-1a in p53null cells compared to p53WT A549 cells. Real-time qPCR analysis of IFITM1, IFITM2, IFITM3, and p21 gene expression in untreated or IFN-β-1a (50unit/ml) treated p53WT A549 cells and p53null A549-KO1, A549-KO2 and A549-KO3 cells at 24 hours post treatment. **p*<0.05. *p* values were calculated on the significance of differences between each different knockout cells and control A549 cells.


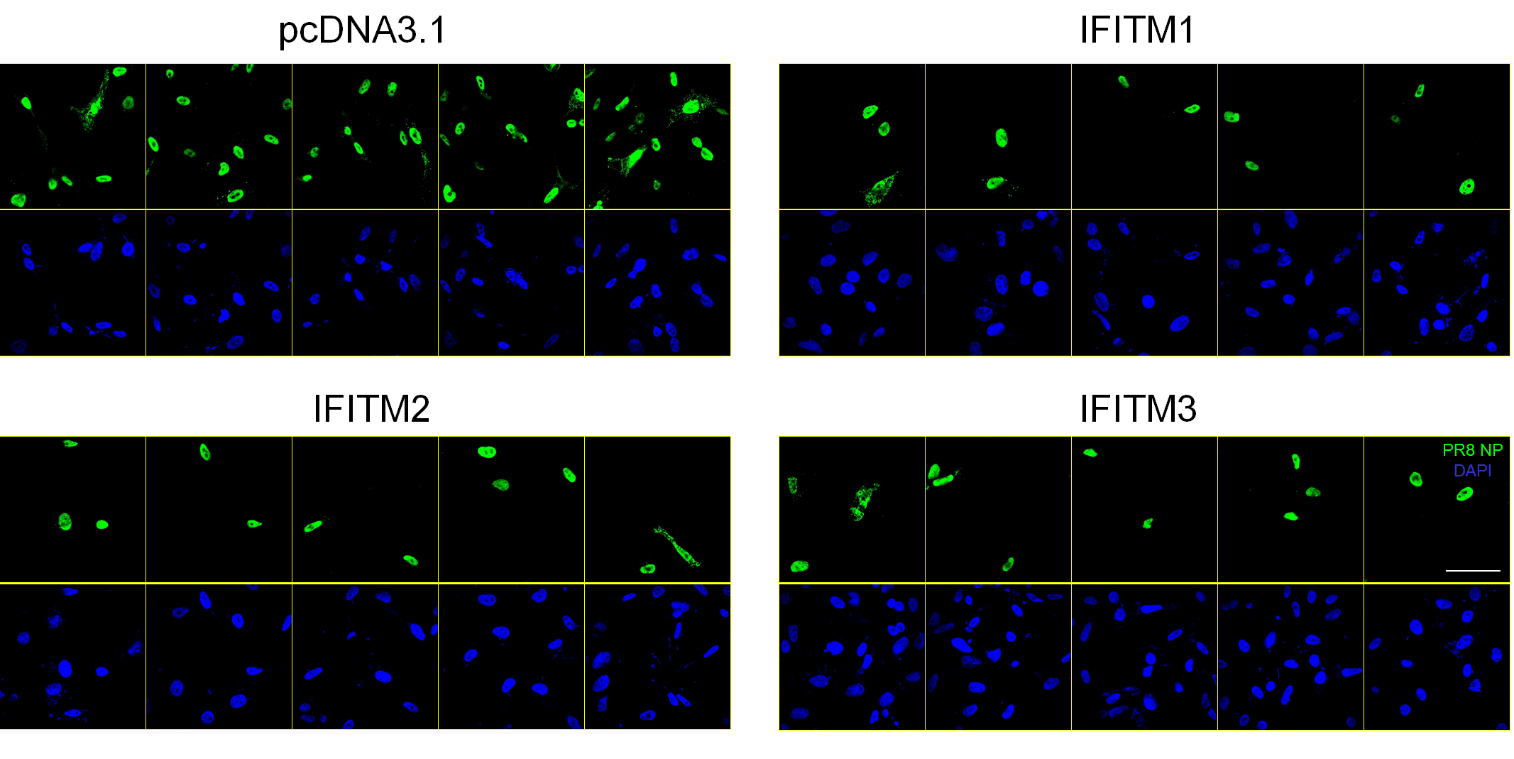


**Figure S6** Fluorescence images of IAV infected A549 cells with overexpressed IFITMs proteins. p53WT A549 cells were transfected with IFITM1, IFITM2, or IFITM3 expression plasmids, or pcDNA3.1 vector control, and 24 hours later were replated and infected with IAV (MOI=0.001). After 24 hours post infection, cells were harvested and labeled for IAV NP (green) with Hoechst 33342 to stain nuclei (blue). For each condition, five areas were randomly selected for image capture. Scale bar: 50µm.


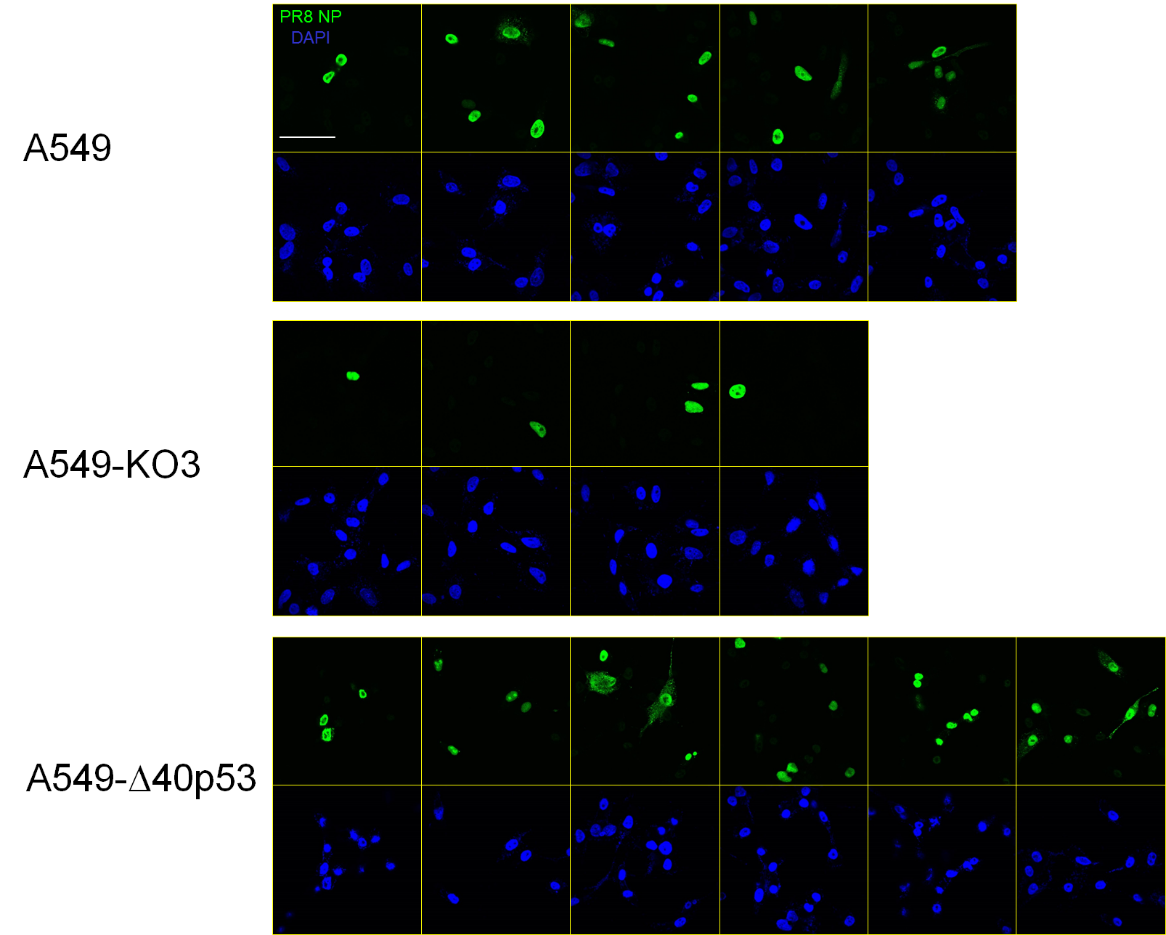


**Figure S7** Fluorescence images of IAV infected A549, A549-KO3 and A549-Δ40 cells. A549, A549-KO3 and A549-Δ40 cells were infected with IAV (MOI=0.001). After 24 hours post infection, cells were harvested and labeled for IAV NP (green) with Hoechst 33342 to stain nuclei (blue). For each condition, four to six areas were randomly selected for image capture. Scale bar: 50µm.
